# Serotype independent protection induced by a vaccine based on the IgM protease of *Streptococcus suis* and proposal for a new immunity-based classification system

**DOI:** 10.1186/s40813-024-00398-2

**Published:** 2024-10-14

**Authors:** A. A. C. Jacobs, A. W. F. Grommen, S. Badbanchi, A. J. van Hout, T. J. van Kasteren-Westerneng, L. Garcia Morales, R. Bron, R. P. A. M. Segers

**Affiliations:** 1 MSD Animal Health, Wim de Körverstraat 35, PO Box 31, Boxmeer, 5830 AA The Netherlands; 2 MSD Animal Health Innovation GmbH, Zur Probstei, 55270 Schwabenheim, Germany; 3 Royal GD, Arnsbergstraat 7, Postbus 9, Deventer, 7400 AA The Netherlands

## Abstract

The IgM protease (*Ide*_*Ssuis*_ gene; Gene ID 8153996) of *Streptococcus suis* is a putative virulence factor that has been shown to be a protective vaccine antigen for pigs (Seele et al. Vaccine 33:2207–12, 2015). To assess its potential as a cross-protective antigen, the amino acid variability among prevalent clinical isolates in various regions and among various serotypes was investigated. Multi-sequence alignment of full-length amino acid sequences of *S. suis* IgM protease, available in the public domain (status Jan-2022) supplemented with in-house sequences, i.e. a total of 1999 sequences, revealed that the IgM protease of *S. suis* clusters into three main evolutionary distinct branches: groups A, B and C. Group A, 82% of the sequences in the database, was associated with clinical isolates of various serotypes. Group B, 6% of the strains in the database, was associated with clinical isolates mainly in the EU and mainly belonging to serotype (st) 9. Group C, 12% of the strains in the database, was largely associated with healthy carrier isolates, i.e. nose or tonsil isolates of various serotypes but in particular with st9 and un-typable strains. Within the groups A, B and C, high levels of amino acid identity were observed (> 75%), whereas between groups A and B, the percentage amino acid identity was approximately 30% and between groups A and C approximately 55%. Experimental *Escherichia coli* expressed recombinant subunit vaccines based on the IgM protease group A sequence of st1 strain B10-99, st2 strain 10 or st7 strain 14009-1, induced serotype independent protection in pigs against challenge with all group A strains tested, i.e. strains of different parts of the phylogenetic tree and of different serotypes including st1, 2, 9 and 14. Protection was observed after vaccination of piglets at 3 and 5 weeks of age and subsequent challenge at 7 weeks but also after vaccination of gilts at 6 and 2 weeks before anticipated parturition and challenge of the offspring up to at least 8 weeks of age. No protection was observed against challenge with st9 strain SZ2000-6264 having group B IgM protease. A recombinant subunit vaccine based on the group B IgM protease sequence, also did not protect against challenge with the homologous group B st9 challenge strain. The results indicate that a vaccine based on a group A IgM protease induces protection against all *S. suis* strains that express the group A IgM protease. Depending on the geographical region such a vaccine is expected to protect against 60–100% of the virulent *S. suis* strains. Since the novel proposed IgM protease classification is highly relevant, a PCR was developed and validated, to be able to classify clinical isolates into IgM protease groups A, B and C and predict the cross-protection that can be expected from a group A based IgM protease vaccine.

**Supplementary Information:**

The online version contains supplementary material available at 10.1186/s40813-024-00398-2.

## Background

*Streptococcus suis* is a major problem in the pig industry and can cause arthritis, peritonitis, pneumonia, meningitis and polyserositis in young piglets shortly after weaning, with peak incidence between 4 and 8 weeks of age. Virtually all pigs are carriers of this opportunistic pathogen and disease can be triggered by a variety of stressors such as weaning, transport, introduction into new groups, change in food, poor air ventilation, overcrowding, and/or viral infections such as PRRSV and influenza virus.

Two different typing systems are being used to characterize *S. suis* strains. The oldest and most commonly used typing system is based on capsular polysaccharides that classifies *S. suis* into serotypes (st). Besides this classification, *S. suis* is also genetically differentiated into sequence types (ST) by multi-locus sequence typing (MLST) [3].

Twenty-nine serotypes have been described for *S. suis* [12].

The serotype distribution of clinical (invasive) isolates in the EU has been studied by different research groups [5, 8, 15]. In these studies, the serotypes 9 and 2 were most frequently isolated, followed by 7 and 1.

Many st9 isolates appeared to belong to ST16 as described by King et al., Schultsz et al. and Willemse et al. [3, 8, 14]. These latter researchers sequenced 124 st9 clinical (invasive) and carrier (tonsil) isolates from the Netherlands to study the capsule locus. Based on the capsule locus, Willemse et al. [14] identified a virulent st9 lineage, mainly associated with ST16, that was distinguishable from carrier subpopulations. This finding led to the development of a *cps9K* PCR that is presumed to distinguish virulent st9 strains from non-virulent strains.

Currently available vaccines (mostly autogenous vaccines) consist of killed whole cells combined with an adjuvant that induce only serotype-specific protection [12]. Given the large number of serotypes, there is a need for a cross-protective vaccine that covers all relevant serotypes.

The first line of host defence against many infections is the production of IgM that can opsonize the respective pathogen, after which it can be further attacked by the host immune system. The ability to degrade IgM antibodies gives a competitive advantage for the pathogen. The IgM protease of *S. suis* (*Ide*_*Ssuis*_ gene, Gene ID 8153996) is a putative virulence factor [9] that has been shown to be a cross-protective vaccine antigen [6, 10].

In this study we further investigated the IgM protease molecule regarding amino acid identities (phylogenetic tree), performed vaccination-challenge studies and introduced a new classification system for *S. suis* based on immunological cross-protection afforded by the IgM protease. In addition, a PCR was designed to classify clinical isolates into IgM protease groups A, B or C.

## Methods

### *S. suis* strains

The *S. suis* strains, clinical pig isolates, that were used as challenge strain and/or for cloning of the IgM protease and/or for validation of the qPCR are shown in Table 1. Further details of the strains can be found in Supplementary data I.

**Table 1:**
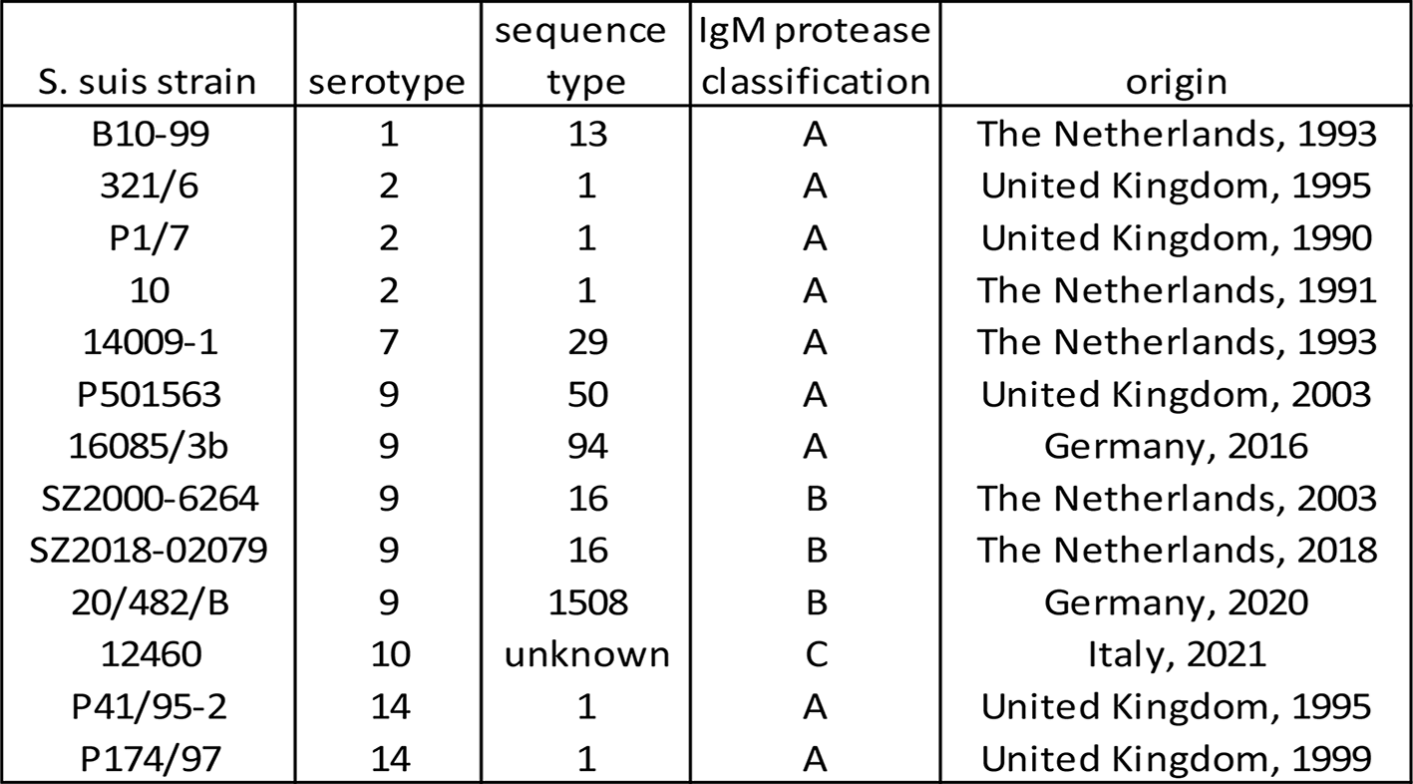
*S. suis* strains used in this study

### Preparation of *S. suis* challenge cultures

Challenge cultures of different *S. suis* strains were prepared by inoculation of bacterial stock on blood agar plates at 37 °C for 20 h and then subcultured in Todd Hewitt Broth supplemented with 0.1% cysteine at 37 °C until the culture was in the exponential growth phase. Subsequently, the challenge culture was diluted to an OD_600_ of 0.15 (st2 strain 321/6 and st2 strain P1/7) or concentrated by centrifugation to OD_600_ of 1.7 (st9, ST16 strain SZ2000-6264) or OD_600_ of 3.5 (st1 strain B10-99, st9 ST50 strain P501563, st14 strain P41/95-2 and st14 strain 174/97) and then transported to the challenge facility on ice. The actual live count of the challenge cultures was determined after use (plate counting).

### rIde constructs and antigen production

The respective IgM protease genes were synthesised to encode 10 C-terminal histidine residues, cloned in *E. coli* expression vector pET-45b(+) and transformed into BL21(DE3) cells. After growth in autoinducing medium the protein was obtained from the supernatant of cell lysate. The recombinant IgM proteases were purified following the method described by Seele et al. [9]. The preparations were inactivated using 0.2% BPL (β-propiolactone) and stored at 4 °C until use.

### Database generation and protein annotation

A database of all publicly available IgM protease sequences was created using the Bio.Entrez package to download *S. suis* assemblies from NCBI as of Jan-2022. A total of 2,899 unique assemblies were obtained from RefSeq and GenBank databases. The same package was also employed to associate the corresponding metadata to each entry, focusing on attributes such as collection date, geographic location, isolation source, strain, and serotype. This database was merged with an internal database of 212 isolates and their corresponding metadata. Prokka (version: 1.14.5) was utilized to annotate all the assemblies, allowing extraction of the IgM protease in 2,440 assemblies from NCBI and 146 from internal entries [11]. The needle tool from EMBOSS (version: 6.6.0), an implementation of the Needleman-Wunsch algorithm, was subsequently used to align each extracted sequence to the reference IgM protease (gapopen 10.0 gapextend 0.5) to identify the sequence Identity [4]. Following this, the gene sequence for each entry was extracted via the gene coordinates from each annotation and the faidx tool from the SAMtools suite (version: 0.1.19, Danecek et al. [1]. In addition, the sequence types were determined by multilocus sequence typing (MLST) using the pyMLST package (version: 2.1.3, Valot [13]. Finally, several web-based platforms InterProScan, PDBSum and PredictProtein were used to annotate the protein, and identify functional domains.

### Vaccines

Four different recombinant IgM protease antigens were used for vaccine preparation: the group A rIde-B10-99, rIde-10 and rIde-14009-1 and the group B rIde-SZ2000-6264 which were derived from the sequences of st1 ST13 strain B10-99, st2 ST1 strain 10, st7 ST29 strain 14009-1 and st9 ST16 strain SZ2000-6264, respectively. The vaccine antigen concentration varied from 50 to 230 µg per 2 ml vaccine dose. For the piglet vaccination-challenge studies, Emulsigen or X-Solve adjuvants were used. Emulsigen is a commercially available o/w adjuvant (MVP Adjuvants, Phibro Animal Health Corporation) that constituted 20% of the final vaccine composition. X-Solve is an in-house oil in water adjuvant consisting of a 5 to 1 v/v blend of a micro-emulsion containing paraffin as the immunogenic oil and a nano-emulsion containing Vitamin E acetate as the immunogenic oil, respectively, and is 1:1 mixed with the antigen. For the sow vaccination studies two different adjuvants were used, X-Solve (as described above) and micro-DF. The latter adjuvant is an in-house nano-emulsion that contains Vitamin E acetate as the immunogenic oil and is 1:1 mixed with the antigen.

### Design of experimental piglet vaccination-challenge studies

All the piglet vaccination-challenge studies had a similar design and consisted of a vaccine group and an unvaccinated control group. Pigslets were obtained from a PRRS negative farm with no recent *S. suis* cases. Treatment group size varied between 10 and 14 piglets and the groups were composed of piglets from different litters (maximally 2 or 3 piglets per litter) to minimize any litter effects. Vaccinations (2 ml dose) were administered intramuscularly at 3 weeks and 5 weeks of age, except for study 1 where vaccine was administered at 5 weeks and 7 weeks of age. On the day of first vaccination and on day of challenge, blood samples (serum) were collected to determine antibody titres against the IgM protease. The piglets were challenged intratracheally at 7 weeks of age except for study 1 where the challenge was performed at 9 weeks of age, using a 10 ml dose of fresh cultures of different strains. The challenge doses were 1 × 10^9^ CFU for strains 321/6 (st2) and P1/7 (st2), 5 × 10^9^ CFU for strain SZ2000-6264 (st9, ST16), 4 × 10^9^ CFU for strain P501563 (st9, ST50), 3 × 10^10^ CFU for strains P41/95-2 (st14) and 174/97 (st14). Post-challenge, the piglets were observed daily for clinical symptoms of polyserositis according to a clinical scoring system. The score system consisted of four parameters, i.e., demeanour, locomotion, respiration, and neurological signs and for each parameter scores 0 = normal, 1 = mild, 2 = moderate, 3 = severe were assigned. The humane endpoint (HEP) was reached if one or more parameters had score 3. The first 4 days post-challenge, the animals were observed at least 3–4 times per 24 h with intervals of maximally 6–8 h. On days 5–11, the observation frequency was maintained or reduced, depending on evolution of clinical signs and as judged by the responsible veterinarian, all aiming at avoidance of severe discomfort and disease related mortality. Animals that reached the humane endpoint were euthanized and necropsied. Blood samples (day 2 post-challenge) and/or swab samples from affected organs, were cultured on blood agar to confirm the infection.

### Design of experimental gilt vaccination-piglet-challenge studies

Two gilt vaccination studies with piglet challenge were performed. Both studies had a similar design and consisted of a vaccine group and unvaccinated control group. The gilts were obtained from a PRRS negative farm with no recent *S. suis* cases. Gilts (5 or 6 per group) were randomized using a stratified randomization. The piglet challenge groups were composed of maximally 2 piglets per gilt to minimize any litter effects. Vaccine (2 ml) was administered intramuscularly at 6 weeks and 2 weeks before anticipated parturition. On the day of first vaccination and on the day of parturition blood samples (serum) were collected to determine antibodies against the IgM protease. In addition, colostrum samples were collected to test for antibodies. The offspring (piglets) were challenged intratracheally at 4 or at 8 weeks of age, using 10 ml of a fresh culture of st1 strain B10-99 (3 × 10^10^ CFU per dose) or st2 strain 321/6 (1 × 10^9^ CFU per dose), respectively. The post-challenge evaluation was done as described above.

### Statistical analysis

For all vaccination-challenge studies disease related culling was the main protection parameter and was analysed in the Fisher’s exact test using Minitab Statistical Software version 21.4.1.

Serological responses were evaluated using descriptive statistics (Microsoft Excel).

### rIde-14009-1 ELISA

The serum and colostrum samples were tested in an ELISA to determine the antibody titre against the IgM protease. For this purpose, the recombinant rIde-14009-1 antigen was coated onto microtiter plates. After coating, the plates were washed, and serial dilutions of samples were made. After incubation and subsequent washing, the bound antibodies were quantified using anti-swine IgG conjugate and TMB as substrate. Titres were expressed in log_2_. In this test the lower detection limit is 4.3 log_2_ or 3.9 log_2_, depending on the predilution of the samples. For calculation purposes < 4.3 and < 3.9 were replaced by 3.3 and 2.9, respectively.

### qPCR assay

To identify strains containing IgM proteases of the different groups, a qualitative qPCR analysis with SYBR green chemistry was developed and optimised. For this purpose, three group-specific primer mixes were designed to anneal to genes encoding for IgM protease group A, B or C. To do so, IgM protease encoding genes from the database (supplementary data I) were aligned, and small conserved regions were identified for primer design. Primer pairs were checked to anneal in > 95% of sequences of the corresponding group and not able to anneal in any sequence of the other groups, while allowing up to 5 miss-matches, but not in the 3’ end. The primer sequences can be found in Table 2. Colony material from strains from group A (strains P501563 and P1/7), group B (strains SZ2018-02079 and 20/482/B) and group C (strain 12460) were used as a positive control for the assay. The optimized PCR protocol was as described below.

**Table 2:** Group-specific oligonucleotides for qPCR classification of IgM protease

Primer mix	Primer names	Sequences
Group A - forward	ideS-A1 ideS-A2	gtg gta ttg agt ctg gtt gtt c gtc aga cac cct aat cgt ag
Group A - reverse	ideS-A3 ideS-A4	gtt tca act aac tct gtc tc atg agc tat cag aat act gtg
Group B - forward	ideS-B1a ideS-B1b ideS-B2	agt ttc ttc caa cat gtt gc agt ttc ttc caa tat gtt gc atc agc tcc ata ttt ctc
Group B - reverse	ideS-B3 ideS-B4a ideS-B4b	gtg aaa aag taa cca ata ac tat tta ata gcc ttt tct ct tat tta ata gct ttt tct ct
Group C - forward	ideS-C1	gca act tga tct tct tct ttt ac
Group C - reverse	ideS-C2	gaa ctt cgt gaa tca gaa ac

Template was prepared by adding colony material to 50 µl WFI and lysed for 5 min at 95 °C. 2 µl lysate was used directly in the qPCR mix as template. The qPCR mix consists of 12.5 µl “SSO Advanced Universal SYBR Green Supermix” (Biorad), 1 pmol of each primer, 2 µl of template and WFI added to a final volume of 25 µl. The qPCR program started with 95⁰C, 5 min (denaturation step), followed by 35 cycles of denaturation at 95⁰C for 10 s, annealing and data collection at 50⁰C for 30 s. The cut off value of the three assays are Ct = 20 (Group A), Ct = 21 (group B) and Ct = 23 (group C).

The PCR was validated following the guidelines of VICH on bioanalytical methods by BaseClear B.V. (Leiden, The Netherlands).

## Results

### Multi-sequence alignment and phylogenetic tree

Publicly available sequences of the IgM protease (status Jan-2022), supplemented with available in-house sequences, were used to generate a database of 2586 sequences. The sequenced isolates were from different geographical regions including the US, Canada, UK, Germany, France, Italy, Spain, The Netherlands, Denmark, Switzerland, Thailand, China, Korea, Japan and Vietnam. A significant part of the sequenced strains was confirmed non-clinical carrier isolates (i.e. tonsil or nasal isolates from healthy animals), whereas confirmed invasive strains are most relevant for vaccine research. Moreover, for many strains the metadata did not indicate whether the isolate was clinical or non-clinical, and the tissue from which the organism was isolated (e.g. brain/blood/joint vs. nasal/tonsil) was missing and therefore were considered non-confirmed clinical and non-confirmed non-clinical isolates.

**Fig. 1:**
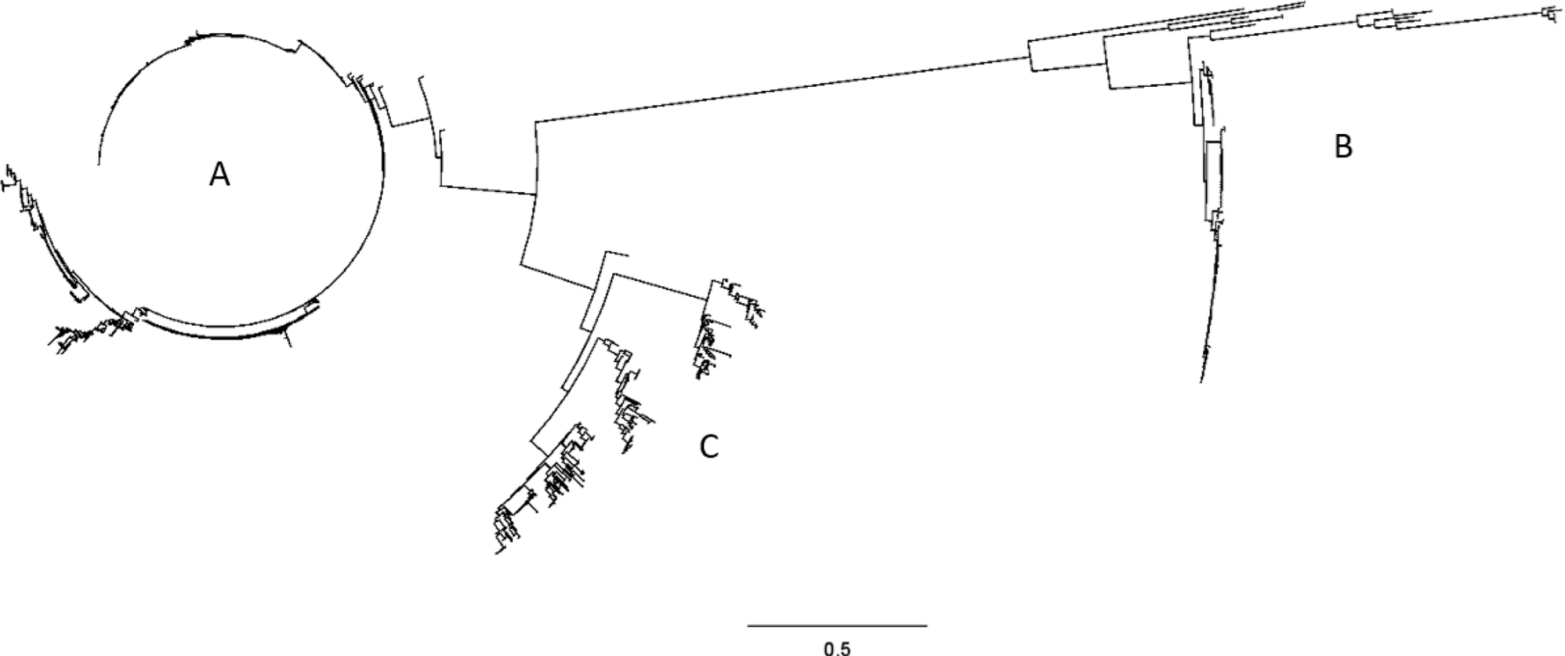
High level phylogenetic tree of S. suis IgM protease based on 1999 sequences. The sequences were aligned using Geneious global alignment with free end gaps (Geneious prime software, version 2021.1.1). This alignment was used to build a phylogenetic tree using the tree building feature of Geneious (tree build method: PhyML; substitution model: le Gascuel)

All IgM protease sequences were translated to full-length amino acid sequences, which consisted of approximately 990–1280 amino acid residues depending on the number of repeats. Duplicate sequences and incomplete sequences with < 800 amino acids were deleted, which resulted in a database of 1999 sequences (Supplementary data I). This database was used for multi-sequence alignment to study the evolutionary relationship and to create a phylogenetic tree. This analysis revealed that the IgM protease of *S. suis* clusters into three main evolutionary distinct branches: groups A, B and C (Fig. 1).

Group A, 82% of sequences in the database, was associated with clinical and non-clinical isolates of various serotypes. If only confirmed clinical isolates are considered, group A represented 91% of the sequences in the database (Table 3).

**Table 3:**
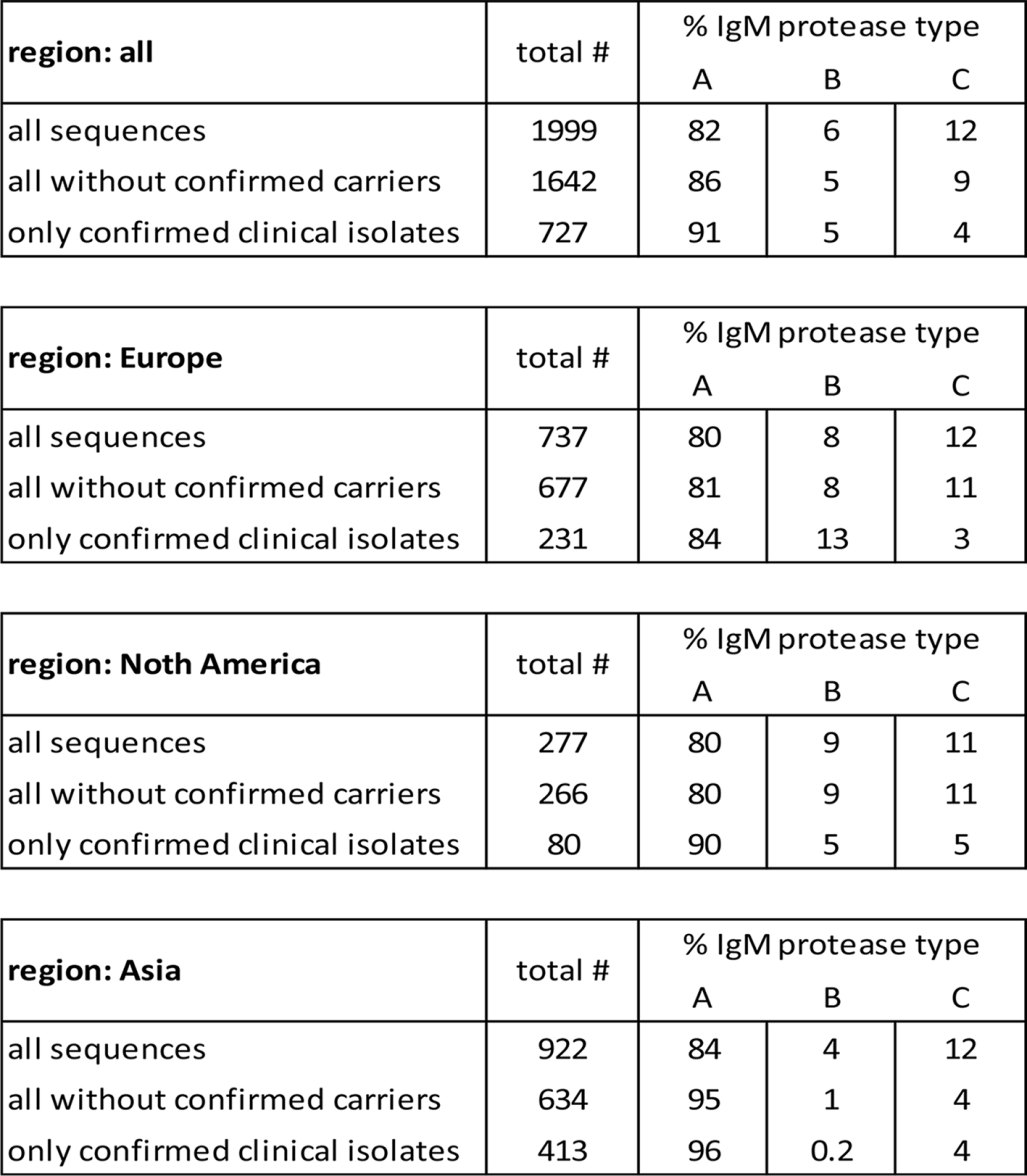
Distribution of IgM protease A, B and C in database, all geographical regions and split per region

Group B, 6% of the sequences in the database, was associated with clinical and non-clinical isolates. If only confirmed clinical isolates are considered, group B represented 5% of the sequences in the database (Table 3). If only EU strains are considered, group B represented 13% of the sequences, indicating that group B is especially prevalent in the EU. These clinical group B strains were mainly associated with st9, particularly st9 of ST16, and to a lesser extent of ST136.

Group C, 12% of the sequences in the database, was predominantly associated with healthy carrier isolates (i.e., nose or tonsil isolates). If only confirmed clinical isolates are considered, group C represented only 4% of the sequences in the database, confirming that this group C IgM protease is relatively more often associated with non-invasive (carrier) isolates (Table 3). Group C IgM protease was largely associated with st9 and un-typable strains and to a lesser extend also with st4, 10, 24 and 31.

**Fig. 2:**
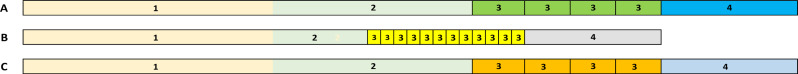
Schematic presentation of *S. suis* IgM protease molecule. Based on multi-sequence alignments the IgM protease can be divided into three main groups **A**, **B** and **C**. Within the IgM protease molecules 4 regions can be identified: Region 1 contains a MAC1 domain with predicted hydrolase activity. Region 2 is linked to structural function and has a sequence linked to substrate binding. Region 3 is a repeat region with predicted casein kinase II phosphorylation site (group **A** and **C**) or N-meristoylation site (group **B**). Region 4 is a trans-membrane region linked to cell wall anchoring. Same color indicates high level of amino acid identity

In other regions i.e. North America and Asia, clinical isolates are dominated by group A IgM protease, whereas group B and C are more often associated with carrier isolates (Table 3). Multi sequence alignments showed that the main differences between groups A, B and C can be found in the C-terminal half of the protein (Fig. 2). Group A IgM protease molecules have a similar structure with a variable number of repeats consisting of approximately 60 amino acids each. Group C IgM protease molecules also have a variable number of nearly identical repeats of approximately 60 amino acids each, which have no similarities to those of group A. Group B IgM protease has a variable number of (short) repeats that consist of 16 amino acids each.

### Sequence similarity search and functional domains

Sequence similarity search using Needleman-Wunsch alignment in addition to protein annotation (PDBSum, InterPro and PROSITE), identified 4 regions in the IgM protease molecule, designated 1–4, as indicated in Fig. 2.

Region 1 (approximately 390 amino acids) contains a MAC1 domain with predicted hydrolase activity. For all groups A, B and C sequences, this is the region with the highest percentage of amino acid identity. The C-terminal half of the IgM protease molecules of groups A, B and C show much less amino acid sequence identity.

Region 2 (approximately 260 amino acids) consists of Alpha helices and intrinsically disordered regions. This segment has been linked to structural functions (e.g. involved in proper folding) and contains a region linked to substrate binding. In addition, the whole region shows similarity (30%) with ATP-binding transferase proteins.

Region 3 consists of repeats that can be characterized as low complexity regions. The repeat regions of groups A and C consist of 1–6 repeats that contain a (PROSITE) predicted Casein kinase II phosphorylation site whereas the group B repeat regions contain a predicted N-myristoylation site. This region has a variable length (approximately 60 to 350 amino acids), depending on the number of repeats.

Region 4 (consisting of approximately 280 amino acids) is a predicted transmembrane segment, indicating a cell wall anchor function. This region contains amino acid motifs and/or sequences with similarities to a GOL-ligand binding site, which is comprised of a transmembrane helix and a nuclear localization signal.

### Vaccination-challenge studies

To test the immunity after vaccination, an intratracheal challenge model was used. In susceptible pigs, this model results in locomotory symptoms and/or severe depression (as a result of poly-serositis in the abdominal and/or thoracic cavity) and seldomly in neurological sigs. Pneumoniae has never been found after challenge (results not shown). In most cases clinical signs progressed to a humane endpoint whereas most protected animals remained free of clinical signs. For all affected pigs, in all studies, the *S. suis* infection was confirmed by either a positive blood culture (2 days post-challenge) and/or a positive swab sample from affected tissue (mostly fibrin deposits), cultured on blood agar.

**Fig. 3:**
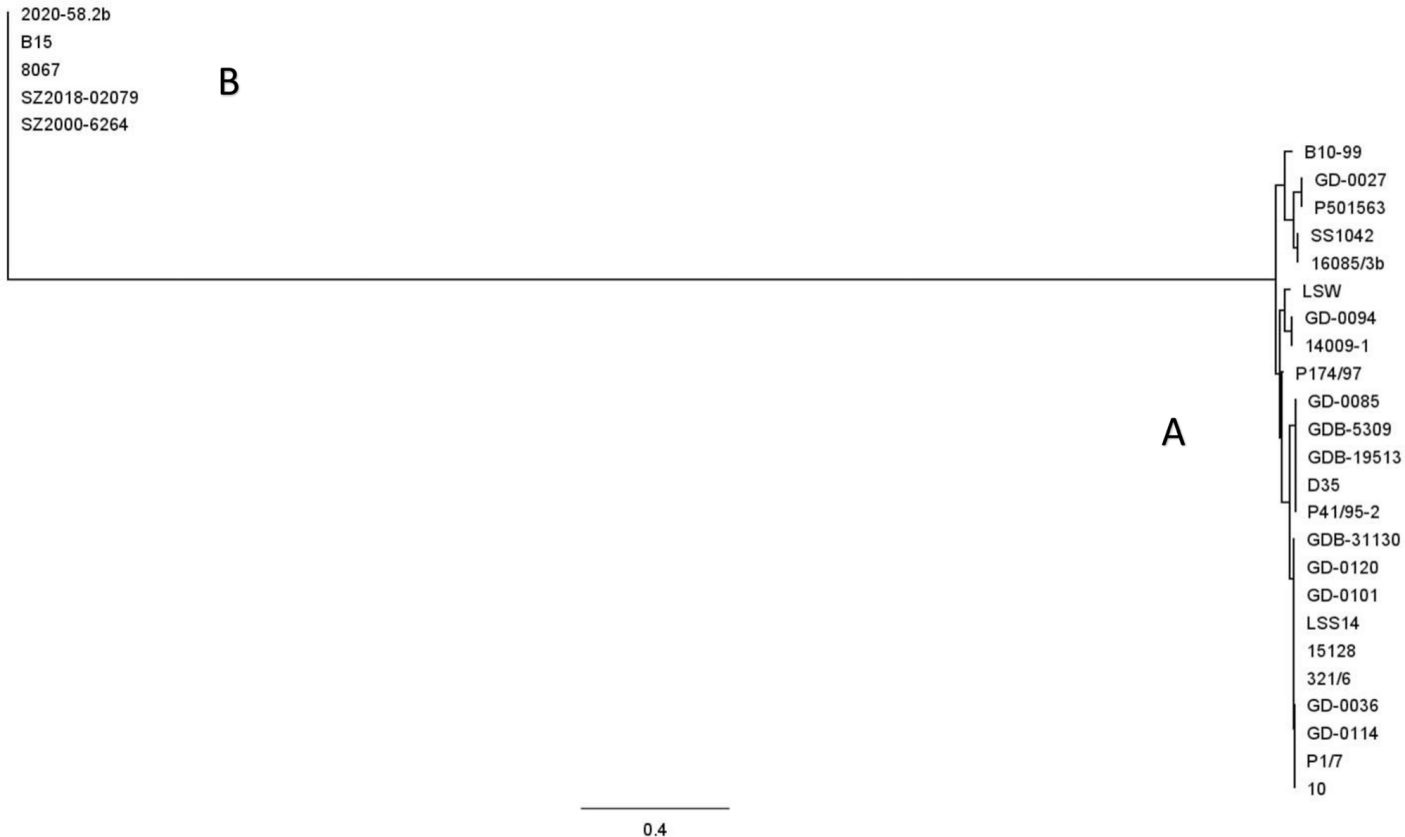
Phylogenetic tree of IgM protease molecules of group A and B of EU clinical isolates. The sequences were aligned using Geneious global alignment with free end gaps (Geneious prime software, version 2021.1.1). This alignment was used to build a phylogenetic tree using the tree building feature of Geneious (tree build method: PhyML; substitution model: le Gascuel)

**Fig. 4:**
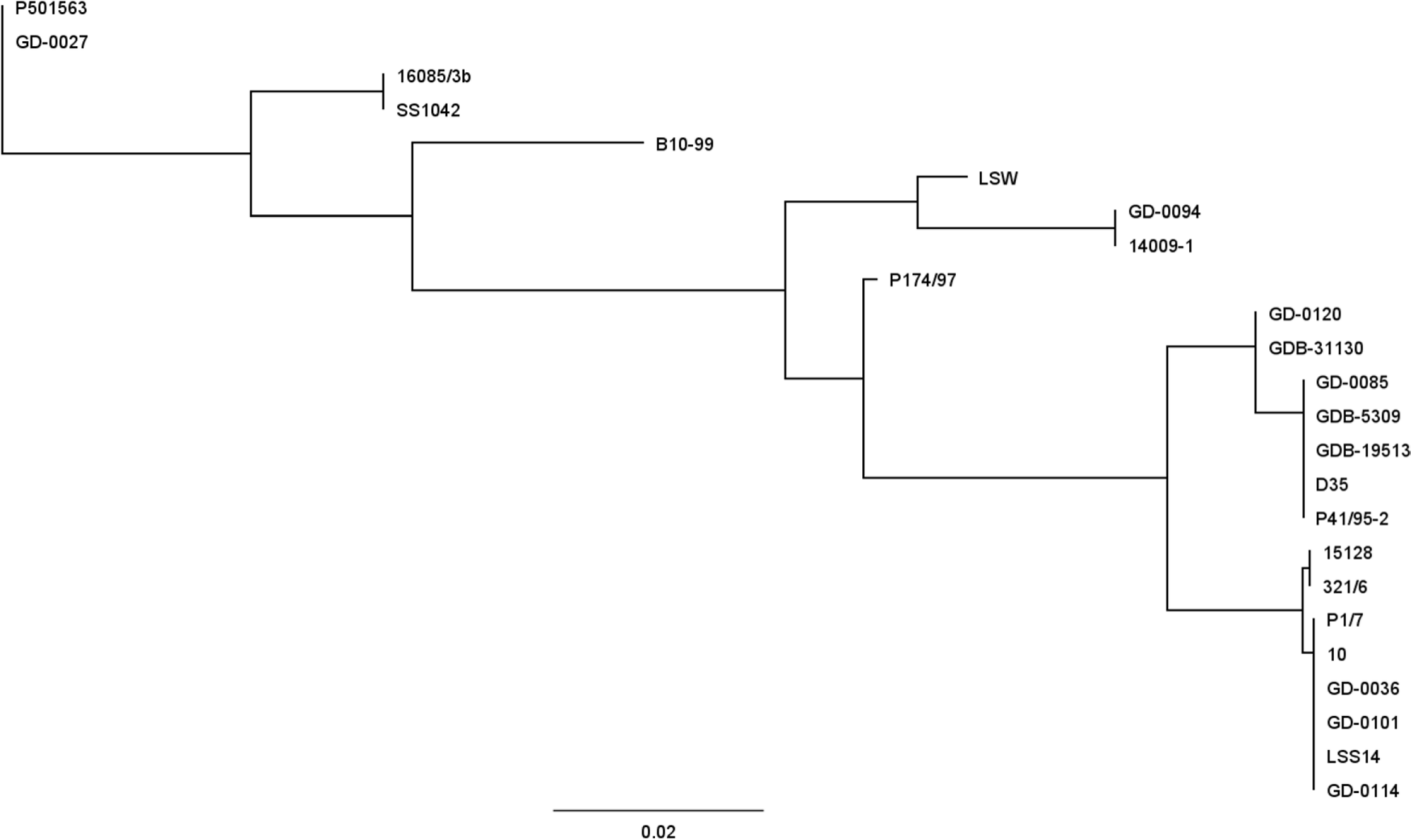
Phylogenetic tree of IgM protease molecules of group A strains shown in Fig. 3. The sequences were aligned using Geneious global alignment with free end gaps (Geneious prime software, version 2021.1.1). This alignment was used to build a phylogenetic tree using the tree building feature of Geneious (tree build method: PhyML; substitution model: le Gascuel)

Since group C IgM protease, appeared to be largely associated with carrier isolates in the EU, our vaccine research was focused on group A and group B. First a phylogenetic tree was created from all groups A and B strains in our strain collection that could be used for infection studies (Fig. 3). In addition, strain 10 (st2) and strain 16085/3b (st9), that were used by other researchers in vaccination-challenge studies, were included as well [6, 10]. The phylogenetic tree confirms the clear separation of groups A and B and the sequence conservation within each of these two groups. The phylogenetic tree considering only group A strains is shown in Fig. 4.

For our research, the IgM protease of group A strains B10-99 (st1), 10 (st2) and 14009-1 (st7) were cloned and expressed in *E. coli*. These recombinant antigens were tested as a vaccine antigen and appeared to induce protection in piglets against mortality caused by challenge with *S. suis* group A strain 321/6 (st2) after both piglet and sow vaccination (Table 4, study 1, 2 and 3, respectively). The sow vaccination study 2 also showed that the duration of the maternally derived immunity is at least 8 weeks.

**Table 4:**
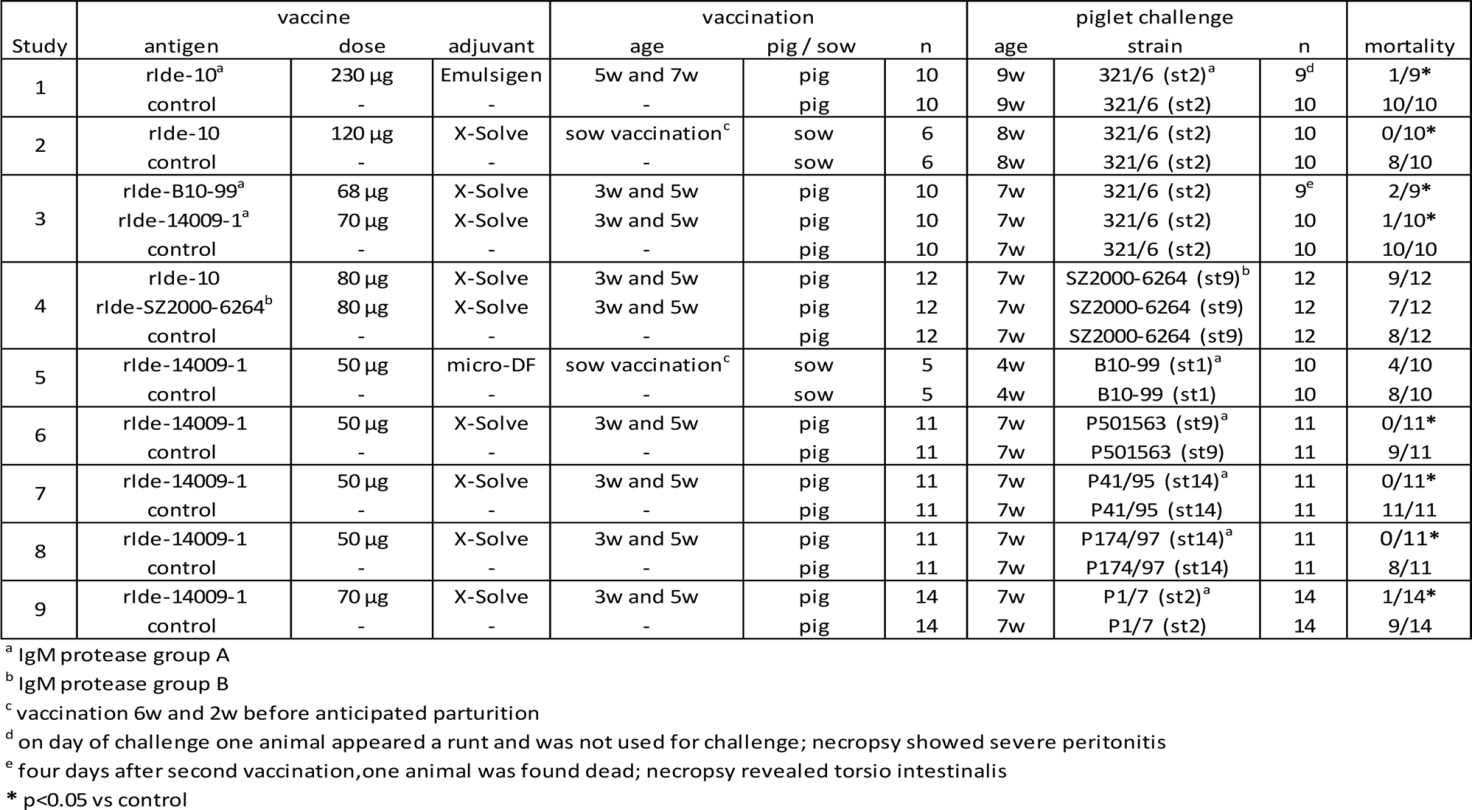
Overview of vaccination-challenge studies in piglets and sows

However, no cross-protection was observed against challenge with *S. suis* st9 ST16 strain SZ2000-6264 belonging to IgM protease group B, and the homologous antigen rIde-SZ2000-6264 also did not induce protection against this strain (Table 4, study 4).

Because strain 14009-1 (st7) is in the middle of the phylogenetic tree with highest degree of similarity with other IgM protease molecules in the tree, the rIde-14009-1 construct was further tested and appeared to induce protection against several different group A challenge strains evenly distributed over the phylogenetic tree (Table 4, studies 5, 6, 7, 8 and 9). The heterologous challenge strains included st1 strain B10-99, st2 strain P1/7, st9 strain P501563, st14 strain P41/95 and st14 strain P174/97.

Immunogenicity of the vaccines used in the vaccination-challenge studies were further evaluated by serological responses. On the day of first vaccination the piglets (3 or 5 weeks of age) had low antibody titres in the range 3.1–5.8 log_2_ (Table 5). After two vaccinations with the group A IgM protease antigens rIde-B10-99, rIde-10 or rIde-14009-1, the titres had strongly increased, ranging 9.2–12.1 log_2_. Virtually no antibody responses to rIde-14009-1 were observed after two vaccinations with the group B IgM protease antigen rIde-SZ2000-6264. In study 4, the antibody titres to rIde-14009-1 on the day of challenge in group 1, 2 and 3 were 11.3, 5.0 and 4.0 log_2_, respectively (Table 5), whereas antibody titres to the homologous rIde-SZ2000-6264 antigen were 10.1, 9.3 and 5.4 log_2_, respectively, (not shown), indicating that antibodies were formed but not reactive against rIde-14009-1.

**Table 5:**
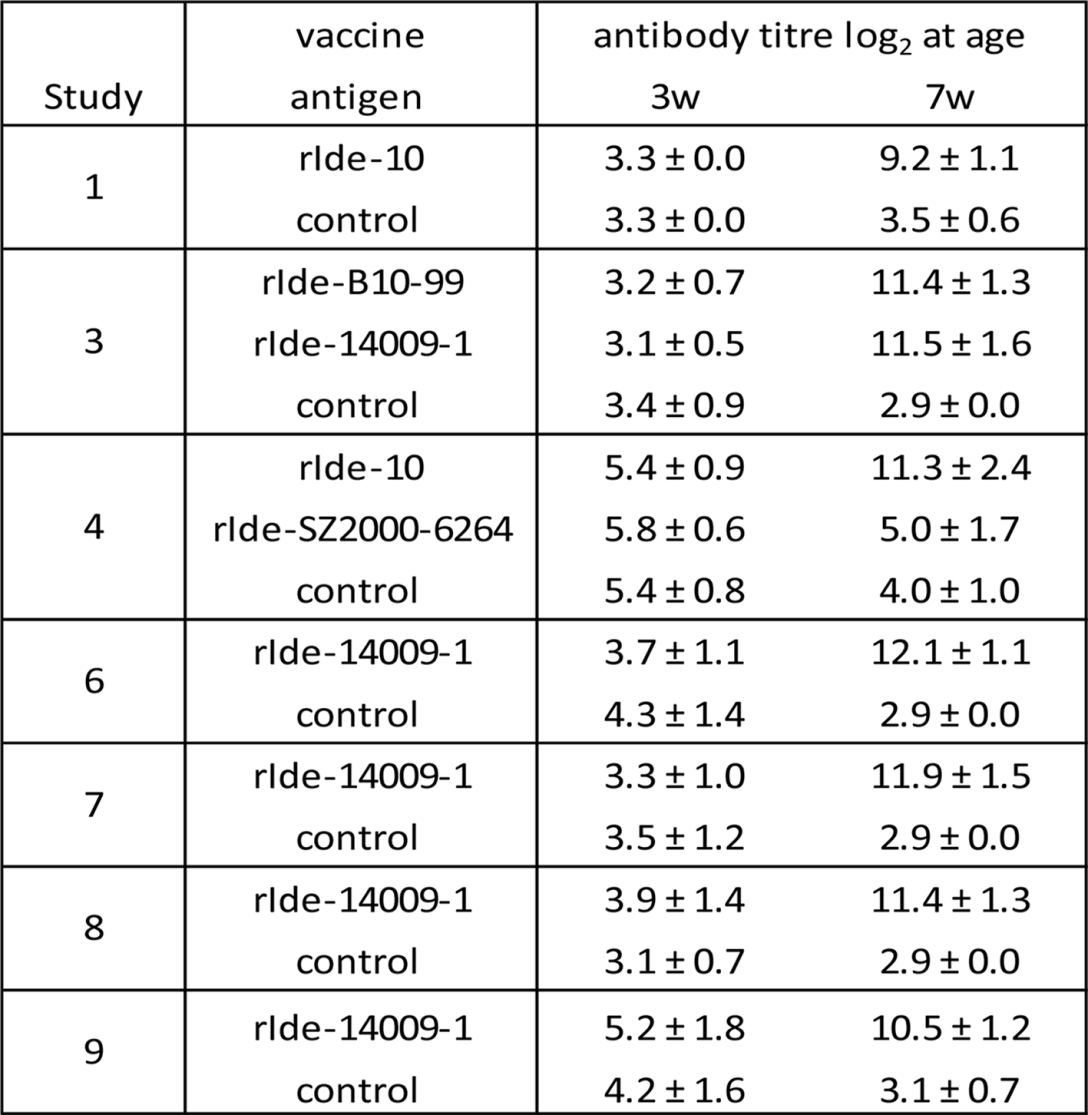
rIde-14009-1 antibody titres in piglet vaccination studies

In sows a similar strong increase in serum antibody titre was observed in the vaccinated groups (from 3.3–5.0 log_2_ to 10.8–12.6 log_2_, respectively) whereas the colostrum titres were approximately 2 log_2_ higher i.e., 13.0 and 14.4 log_2_, respectively (Table 6). The average serum antibody titre of the offspring at 8 weeks of age (day of challenge) was still clearly above the antibody level found in the control animals (8.1 vs. 3.3 log_2_).

**Table 6:**
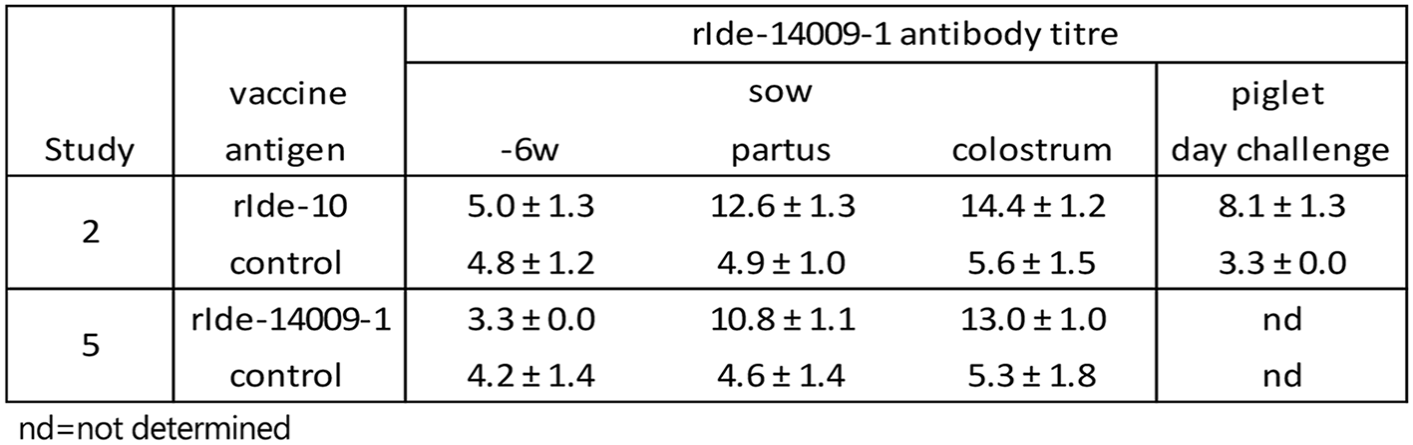
rIde-14009-1 antibody titres in sow vaccination studies

### Additional investigation of serotype 9 strains

From the vaccination-challenge studies it appeared that st9 strains that express group B IgM protease form a distinct group within the species of *S. suis*. Therefore, this group of strains was further investigated.

Willemse et al. [14] have sequenced 124 st9 clinical (invasive) and carrier (tonsil) isolates from the Netherlands to study the capsule locus. After downloading the genome sequences (www.ebi.ac.uk.ena/browser/view/PRJEB20548?show=reads) we were able to extract the IgM protease sequence from 96 of 124 genomes. These sequences were obtained after Jan-2022 and therefore not part of the database. The strain distribution of both clinical and carrier isolates showed that 9 strains belonged to group A, 44 strains to group B and 43 strains to group C (Table 7). All 9 group A isolates and most group B isolates (40/44) were associated with clinical isolates whereas the group C isolates (except 1 strain) were exclusively associated with carrier isolates. Further analysis showed that 39/96 strains were of ST16, all associated with disease; 33/39 ST16 strains had group B IgM protease whereas 6 had group A IgM protease; 7/96 strains were of ST136, had group B IgM protease and all 7 associated with disease.

**Table 7:**
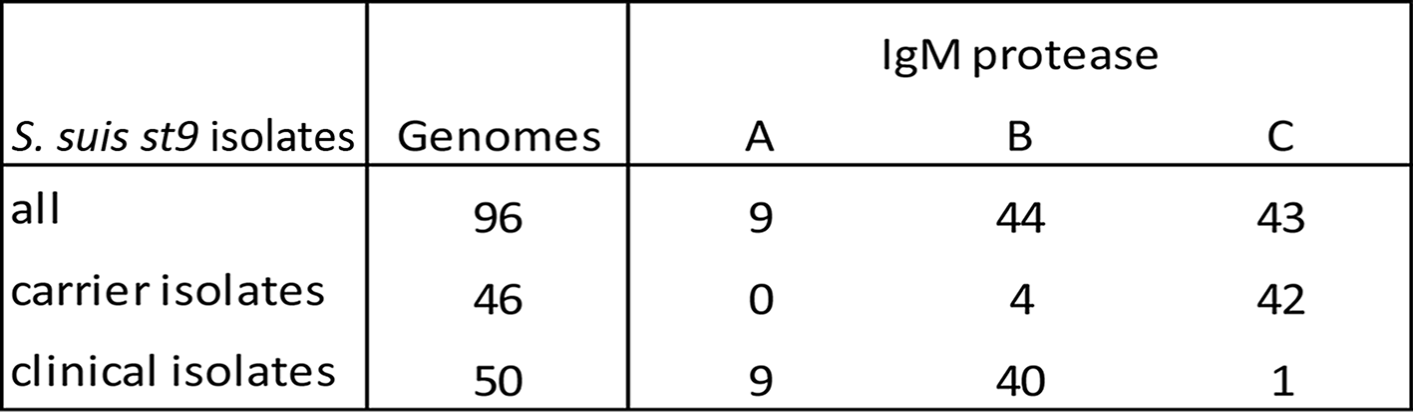
IgM protease distribution among 96 st9 isolates [14] from the Netherlands including clinical and carrier isolates

Additional information on st9 strains was obtained from Zheng et al. [16] who sequenced seven invasive st9 strains from Spain (https://www.ncbi.nlm.nih.gov/). After downloading and translating the IgM protease sequences, it appeared that all seven strains had group A IgM protease.

### Design and validation of a group specific qPCR test

To identify the IgM protease group in *S. suis* isolates, a qPCR test was designed, optimized and validated following the guidelines of VICH on bioanalytical methods. A complete validation report can be found in supplementary data II. Briefly, the designed test was able to correctly classify 10 colonies from each group A (strains P501563 and P1/7), group B (strains SZ2018-02079 and 20/482/B) and group C (strain 12460) over 6 different runs by 2 different laboratory technicians and using different equipment showing good repeatability and precision. Deliberate variations in the concentration of the qPCR components (SsoAdvanced™ Universal SYBR^®^ Green Supermix and primer mix) did not produce a significant increase in Ct values, indicating the robustness of the test. Similarly, serial dilutions of the colony template in nuclease-free water from 1:5 to 1:125 showed no matrix effect and therefore no increase in false negatives. The assay specificity was tested by using colonies of closely related bacteria e.g. *Streptococcus pyogenes*, *Streptococcus equi*, *Streptococcus agalactiae*, *Streptococcus uberis*, *Streptococcus zooepidemicus* and *Glasserella parasuis*, which all showed no amplification.

The statistical analysis of the validation results showed that the cut-off value for a positive outcome in the group A qPCR test is a Ct < 20 (99% confidence), in the group B qPCR test is a Ct < 21 (99% confidence) and in the group C qPCR test is a Ct < 23 (99% confidence). In all, these results show that the 3 qPCR tests here designed and validated, constitute a robust and useful assay to identify the group classification of *S. suis* isolates based on their IgM protease.

## Discussion

Seele et al. [10] and Rieckmann et al. [6] showed that the IgM protease of *S. suis* is a virulence factor as well as a potential vaccine antigen. These researchers found that a vaccine based on a recombinant IgM protease derived from the sequence of strain 10 induced protection against challenge with strain 10 (st2) and strain 16085/3b (st9). In our study we further investigated the IgM protease by using bioinformatics and showed that the IgM protease clusters into three main evolutionary distinct branches: groups A, B and C. We further found that the group A IgM protease can be used as a vaccine antigen that induces serotype-independent cross-protection against all strains that express the group A IgM protease, but no protection was found against a st9 strain expressing group B IgM protease. The group B IgM protease appeared largely associated with strains of st9. In this context it is interesting to note that Rieckmann et al. [6] found protection in pigs, after vaccination with a group A IgM protease-based vaccine, against challenge with st9 strain 16085/3b. We found that this st9 strain belongs to IgM protease group A, and thus, the results of Rieckmann et al. are in line with our results.

Although colostrum intake may highly vary among piglets, especially in large litters, our sow vaccination studies clearly show that maternally derived protection is conferred to the piglets and may indicate an antibody-based mechanism. However, the protective mechanism is currently unknown and is not simply explainable by neutralization of a virulence factor as experimental infection studies by Rungelrath et al. [7] showed no significant difference in virulence between *S. suis* wildtype and isogenic mutants without IgM cleavage activity. In addition, Seele et al. [10] showed that although vaccination with group A rIde_Ssuis_ is associated with killing of *S. suis* in vitro, the isogenic mutant survived or even proliferated in the same piglets.

The lack of protection against group B strains (study 4) may be related to the lack of immunological cross-reactivity and can be explained by the low percentage amino acid identity of approximately 30% between group A and group B molecules versus 75–100% identity within group A. The fact that a vaccine based on rIde-SZ2000-6264 (group B) did not protect against challenge with the homologous challenge strain, despite the presence of homologous antibodies, may indicate that group B strains, in particular those of st9 ST16, have a different virulence mechanism and other antigens may be required for protection. In this context it is relevant to mention that the functionality of the group B and C molecules has not been demonstrated and therefore should be called IgM protease-like molecules rather than IgM protease. In addition, expression of the IgM protease-like protein by group B strains (including challenge strain SZ2000-6264), in vivo, has not been demonstrated. This also could be the reason for the lack of protection.

When analysing the database (Supplementary data I) and considering only confirmed clinical isolates, worldwide, 91% of the isolates have group A IgM protease and are predicted to be protected by a group A IgM protease-based vaccine. In the EU, the vaccine coverage is expected to be slightly lower (84%) as st9 ST16 group B is more prevalent, especially in the Netherlands. According to the latest published serotype distribution results, 49% of the clinical isolates found in the Netherlands is st9 [2], https://issuu.com/gezondheidsdienstvoordieren/docs/varken_99_april_2022). This, in combination with the finding that 82% of the clinical st9 strains have group B IgM protease, implies that approximately 40% of the invasive strains in the Netherlands have group B IgM protease. The occurrence of group B IgM protease in clinical isolates from Germany and Denmark is currently estimated at 15-20% and the group B IgM protease is virtually absent from strains in Italy and Spain (unpublished results). More information regarding this subject was obtained from the study of Zheng et al. [16] who sequenced seven invasive st9 strains from Spain. Further analysing these sequences showed that all seven strains had group A IgM protease, which also indicates that group B IgM protease is very rare in Spain and implying that a vaccine containing group A IgM protease is expected to protect against nearly all strains circulating in Spain.

Based on the sequences in the database (Supplementary data I), group C IgM protease was largely associated with carrier isolates. Further analysing the sequences as published by Willemse et al. [14] confirmed that group C IgM protease, in most cases, is not associated with disease and that vaccine protection against strains having group C IgM protease, especially in the EU, is of low(er) importance. The four st9 ST16 group B strains that were isolated from the tonsils of healthy pigs in that study, could be virulent and accidently isolated from healthy animals. Indeed, these authors mentioned that there were disease outbreaks caused by st9, in the respective farms after study end. If this assumption is correct, the distribution results for the strains of Willemse et al. [14] are even more clearly demarcated, i.e., all IgM protease group A and B strains were virulent and all group C strains, except one, avirulent. Although most st9 ST16 strains (33/39) were associated with group B IgM protease, it is interesting to note that 6/39 ST16 strains had group A IgM protease and thus would be covered by a group A IgM protease-based vaccine. This also shows that ST16 is not exclusively associated with the group B IgM protease.

In this work, a qPCR method was developed to identify *S. suis* isolates in the three different groups according to their IgM protease encoding genes. The assay was validated following the VICH guidelines and was found to be statistically robust and specific. This test facilitates the surveillance of *S. suis* according to the IgM protease group as an additional molecular characterization without the need of genome sequencing. Given that our results indicate good protection conferred by the IgM proteases from group A to *S. suis* isolates containing IgM protease from this group, we propose this tool, and in particular the group A qPCR test, to predict the efficacy of such vaccination to control an *S. suis* outbreak.

## Electronic supplementary material

Below is the link to the electronic supplementary material.

Supplementary Material 1

Supplementary Material 2

## Data Availability

No datasets were generated or analysed during the current study.
