# Supplementary material for: Serotype independent protection induced by a vaccine based on the IgM protease of Streptococcus suis and proposal for a new immunity-based classification system
Source: Porcine Health Manag. 2024 Oct 14;10:41. doi: 10.1186/s40813-024-00398-2 (PMC11472559; doi:10.1186/s40813-024-00398-2)
Supplement: Supplementary file 2 — Supplementary Material 2 [file 40813_2024_398_MOESM2_ESM.pdf]

Validation of *Streptococcus suis* group specific qualitative qPCR

Dr. Mubanga Kabwe, Mirna Baak, Dr. Eline S. Klaassens

BaseClear B.V., Sylviusweg 74, 2333 BE, Leiden, The Netherlands, info@baseclear.com | www.baseclear.com

1. Introduction

BaseClear was requested to perform an inhouse validation of a *Streptococcus suis* group specific quantitative polymerase chain reaction (qPCR) in line with the VICH GL 2 guidelines (www.ema.europa.eu). For the validation of this Q-PCR the following parameters were investigated: precision, specificity and matrix effect. For the analysis, an optimized polymerase chain reaction (PCR) method was used.

2. Materials and methods

2.1 samples

The assays were used to discriminate between the presence and the absence of the *IgM* protease clustered into three distinct branches: A, B and C gene. The respective qPCR primers were designed and supplied by MSD Animal Health Discovery and Technology. The sample material in the form of colonies plated on blood agar were also supplied by the client (Table 1).

Table 1. Test samples.

**Samples**		**Quantity**
Primer mix A forward		5 ml
Primer mix A reverse		5 ml
Primer mix B forward		5 ml
Primer mix B reverse		5 ml
Primer mix C forward		5 ml
Primer mix C reverse		5 ml
***S. suis* group A**	**A1**- P501563	1 agar plate
	**A2**- P1_7	1 agar plate
***S. suis* group B**	**B1**-SZ2018_02079	1 agar plate
	**B2**-20_482_02079	1 agar plate
***S. suis* group C**	**C**-12460	1 agar plate

2.2 qPCR method

In brief, colony material (not more than one week old) was subjected to a qualitative qPCR analysis with SYBR green chemistry using the SsoAdvanced™ Universal SYBR® Green Supermix (BIO-RAD). The qPCR reactions were carried out in the Applied Biosystems 384-well QuantStudio™ 5 Real-Time PCR system (Thermo Fisher Scientific).

The qPCR program started with 95^{0}C, 5 min (denaturation step), followed by 40 cycles of denaturation at 95^{0}C for 10 s, annealing at 50^{0}C for 30 s. To check the specificity of the qPCR amplification and determine the melting temperature (Tm) of the qPCR product, the qPCR method also included a melt curve step with a denaturing step including a 1.94°C/s increase per cycle starting at 95°C for 15 s, an annealing/extension stage at 50^{0}C for 1 min, a melting curve stage at 95°C for 30 s and the cooling stage at 50^{0}C for 15 s.

2.3 Optimisation of the assay

The assay was optimized with regard to the concentration of SsoAdvanced™ Universal SYBR® Green Supermix (BIO-RAD). The optimal qPCR mix concentration was tested between 0.5x, 0.75x, 1x, 1.25x and 1.5x concentration. The same was done for the primer concentrations between 0.5x, 1x and 1.5x (Table 2).

Table 2. qPCR optimization reactions set up.

0.5x Mix:	0.75x Mix:	1x Mix:	1.25x Mix:	1.5x Mix:	
6.3 ul SSO Adv Sybr mix	9.4 ul SSO Adv Sybr mix	12.5 ul SSO Adv Sybr mix	15.6 ul SSO Adv Sybr mix	18.7 ul SSO Adv Sybr mix	
0.5 ul #F primer (10uM)	0.5 ul #F primer (10uM)	0.5 ul #F primer (10uM)	0.5 ul #F primer (10uM)	0.5 ul #F primer (10uM)	
0.5 ul #R primer (10uM)	0.5 ul #R primer (10uM)	0.5 ul #R primer (10uM)	0.5 ul #R primer (10uM)	0.5 ul #R primer (10uM)	**0.5 x primer mix**
2.0 ul template	2.0 ul template	2.0 ul template	2.0 ul template	2.0 ul template	
15.7 ul ddH20	12.6 ul ddH20	9.5 ul ddH20	6.4 ul ddH20	3.3 ul ddH20	
[illegible]tal Volume	*25 ul total Volume*	*25 ul total Volume*	*25 ul total Volume*	*25 ul total Volume*	
**0.5x Mix:**	**0.75x Mix:**	**1x Mix:**	**1.25x Mix:**	**1.5x Mix:**	
6.3 ul SSO Adv Sybr mix	9.4 ul SSO Adv Sybr mix	12.5 ul SSO Adv Sybr mix	15.6 ul SSO Adv Sybr mix	18.7 ul SSO Adv Sybr mix	
1.0 ul #F primer (10uM)	1.0 ul #F primer (10uM)	1.0 ul #F primer (10uM)	1.0 ul #F primer (10uM)	1.0 ul #F primer (10uM)	
1.0 ul #R primer (10uM)	1.0 ul #R primer (10uM)	1.0 ul #R primer (10uM)	1.0 ul #R primer (10uM)	1.0 ul #R primer (10uM)	**1.0 x primer mix**
2.0 ul template	2.0 ul template	2.0 ul template	2.0 ul template	2.0 ul template	
14.7 ul ddH20	11.6 ul ddH20	8.5 ul ddH20	5.4 ul ddH20	2.3 ul ddH20	
25 ul total Volume	*25 ul total Volume*	*25 ul total Volume*	*25 ul total Volume*	*25 ul total Volume*	
**0.5x Mix:**	**0.75x Mix:**	**1x Mix:**	**1.25x Mix:**	**1.5x Mix:**	
6.3 ul SSO Adv Sybr mix	9.4 ul SSO Adv Sybr mix	12.5 ul SSO Adv Sybr mix	15.6 ul SSO Adv Sybr mix	18.7 ul SSO Adv Sybr mix	
1.5 ul #F primer (10uM)	1.5 ul #F primer (10uM)	1.5 ul #F primer (10uM)	1.5 ul #F primer (10uM)	1.5 ul #F primer (10uM)	
1.5 ul #R primer (10uM)	1.5 ul #R primer (10uM)	1.5 ul #R primer (10uM)	1.5 ul #R primer (10uM)	1.5 ul #R primer (10uM)	**1.5 x primer mix**
2.0 ul template	2.0 ul template	2.0 ul template	2.0 ul template	2.0 ul template	
13.7 ul ddH20	10.6 ul ddH20	7.5 ul ddH20	4.4 ul ddH20	1.3 ul ddH20	
25 ul total Volume	*25 ul total Volume*	*25 ul total Volume*	*25 ul total Volume*	*25 ul total Volume*	

2.4 Matrix effect

Due to the possibility that the matrix (colony material) can influence the outcome of the qPCR results, matrix effect was tested in a dilution series. The colony material was prepared by dissolving a loop of colony (1 µL inoculating loop) material in 50 µL of water for injection (WFI) (ThermoFisher Scientific). The suspension was boiled for 5 min at 95°C.

For the Matrix effect analysis. The colony material was resuspended in WFI in four dilutions (undiluted, 1:5, 1:25 and 1:125) and tested in the qPCR assays.

2.5 Specificity of the assay

The assay was tested for specificity against several closely related bacteria eg. *S. pyogenes, S. equi, S. agalactiae, S. uberis, S. zooepidemicus* and *Haemophilis parasuis*. To confirm the specificity of the qPCR for the IgM protease gene of *Streptococcus suis*, the nucleotide sequence of the primers were compared to all available nucleotide sequences using a search engine.

2.6 Validation of the assays

2.6.1 Precision

Repeatability (intra-assay precision) expresses the precision under the same operating conditions over a short interval of time. Intermediate precision (inter-assay precision) expresses variations due to testing on different days, testing by different technicians.

Both repeatability and intermediate precision were determined for the method, including qPCR analysis on 10 colonies by 2 technicians on 6 different runs (i.e. days). Colony material from the *S. suis* strain containing the *IgM* protease clustered into the three distinct branches: A, B and C was used.

2.6.2 Statistical determination of the cut off value

The cut off value of this assay was determined with the precision data. This was calculated with the help of the distribution analysis and estimated tolerance interval (99%).

Statistics were calculated using R software version v3.6.0 with additional packages ‘ggplot2’, ‘tolerance’, ‘rptR’, ‘lmerTest’ and ‘lme4’.

The following calculations were executed; the sigma value with function ‘sd’. The mu value with function ‘mean’. A normal distribution with function ‘dnorm’ with arguments sd and mean. The interval with a confidence of 0.99 with function ‘normtol.int’ with alpha 0.01 and P 0.99.

The estimated %upper limit was calculated with function ‘pnorm’ and mean and sd arguments, with Observed % upper limits as number of items above upper limit divided by total number of observations. Whereas, CPK values were calculated as mu minus upper limit value divided by sigma and then divided by three. Quantiles were calculated with function qnorm and quantile. A two sided tolerance interval was also calculated for 90, 95 and 99% with function ‘normtol.int’ and alpha values of 0.01, 0.5 and 0.1.

Anova calculations were performed with function ‘aov’ with formula Result ~ Technician * Day-Technician.

Results were visualized in plots with ggplot and arguments geom_histogram, geom_vline, annotate and stat_function.

Finally, a Linear mixed model was generated with function ‘lmer’ with the formula: lmer(Result ~ Technician + (1|Day), data = data, REML=TRUE). Thereafter, Anova statistics of this model was calculated with the ‘anova’ function and random effects of the model with function ‘rand’. Least square means tables were generated with function ‘difflsmeans’ with the model and test.effs=”Technician”.

The results can be found in the Addendum 2.

3. Results and discussion

3.1 Optimization of the assay

The results for optimization with regard to the concentration of SsoAdvanced™ Universal SYBR® Green Supermix (BIO-RAD) and the primer mix are shown in Figure. 1 A-C. For Sso Advanced SYBR green Mix, the 1x concentration is in a constant region of low Ct values for all templates. With regard to the primer concentration, a concentration of 1x primer revealed a low Ct value in a relatively constant region and were therefore chosen for subsequent validation analysis. These concentrations are indicated by arrows.

A

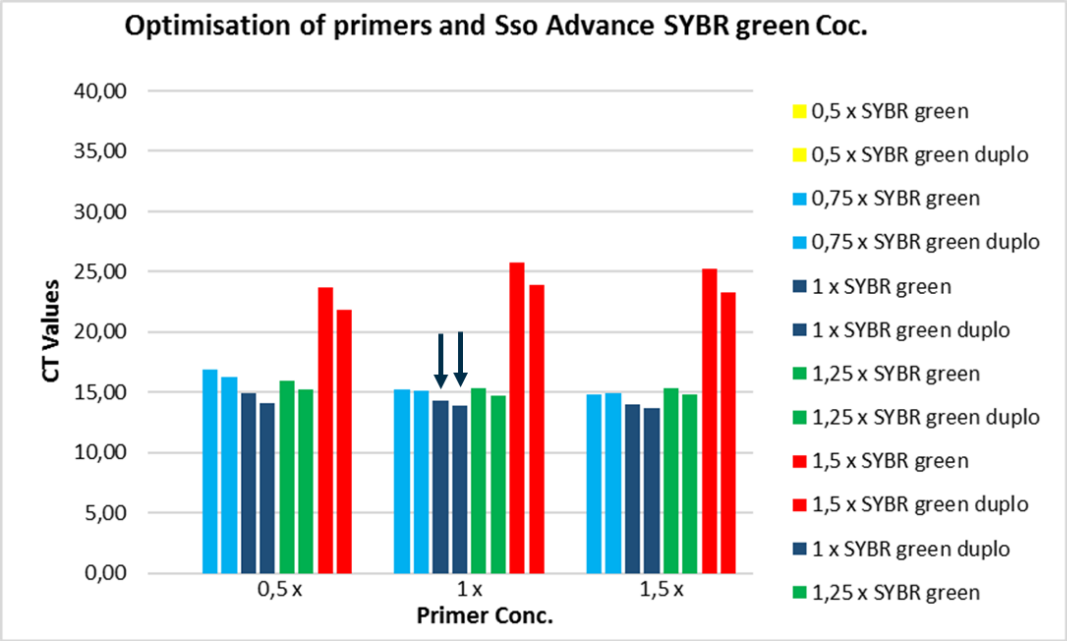

B

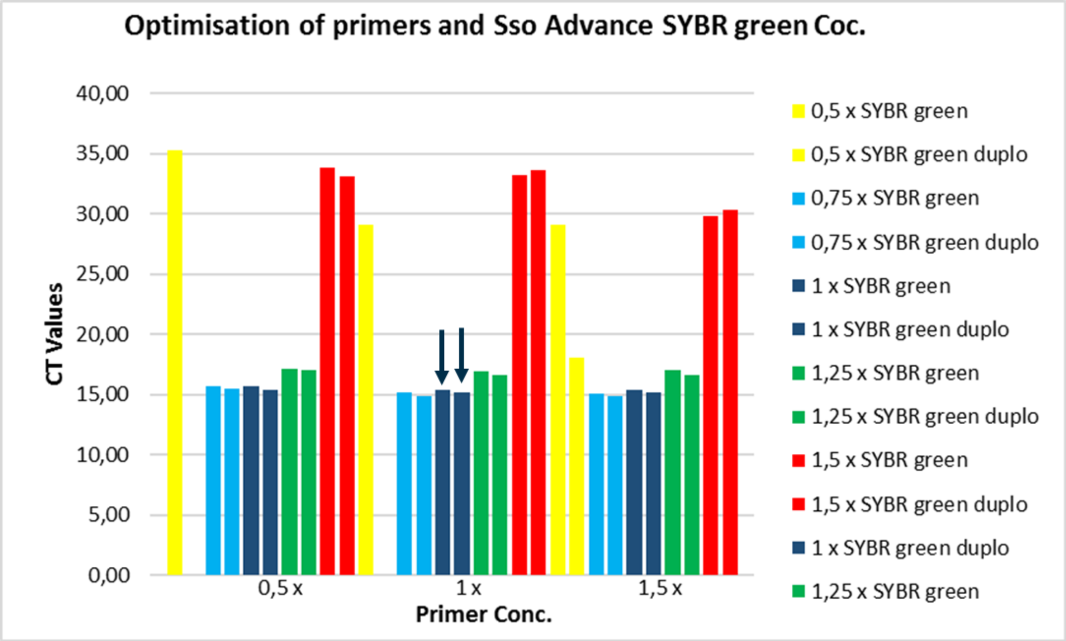

C

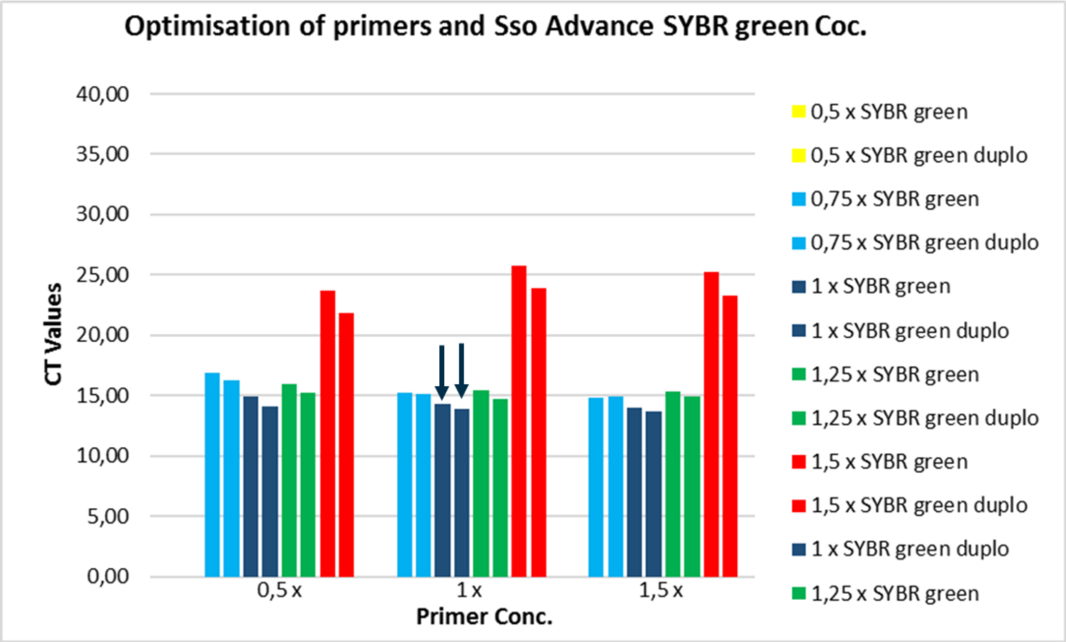

Figure 1. Optimization of the Sso Advanced SYBR green mix and the primer concentration for. A). *S. suis* Group A. B). *S. suis* Group B. C). *S. suis* Group C.

Final /148500 / 1.0

3.2 Matrix effect

The matrix (colony material) was diluted in WFI and tested on possible influence of the outcome of the qPCR results. By testing a series of diluted samples in PCR, no inhibitory effect was seen in any of the diluted DNA samples (Table 3 A - C). All samples were identified as expected for the undiluted samples and sample dilutions of 1:5 to 1:125 there was no risk of false negative results. (Ct<22.0 is positive).

Table 3. Matrix effect (Ct values) for *S. suis* group A, B and C. NTC No Template Control.

Target	Undilute	1:5	1:25	1:125
S. suis A	14,99	16,01	17,83	19,97
NTC	Undetermined			
S. suis B	15,86	17,23	19,28	21,49
NTC	Undetermined			
S. suis C	17,22	18,88	21,08	23,25
NTC	Undetermined			

3.3 Specificity of the assay

The assay was tested against several closely related bacteria and was shown to be specific for IgM protease. Specificity was also confirmed by comparison of all available nucleotide sequences using a search engine. The primers of this assay showed no 100% homology with any sequence of relevant bacteria, except the target sequence.

3.4 Validation of the assay

3.4.1 Repeatability and precision

Both repeatability and intermediate precision was determined for the method. In total 6 different runs on 10 different colonies were done each by two technicians. All samples tested were correctly identified as stated in Table 4 A-C . Full data is shown in addendum 1.

Table 4. Repeatability and precision analysis for *S. suis* group A, B and C.

A

PCR	Samples tested	samples correctly identified	Day	Technician
Group A	10	10	1	1
Group A	10	10	1	1
Group A	10	10	1	1
Group A	10	10	1	1
Group A	10	10	1	1
Group A	10	10	1	1
Group A	10	10	1	1
Group A	10	10	1	1
Group A	10	10	1	1
Group A	10	10	1	1
Group A	10	10	1	2
Group A	10	10	1	2
Group A	10	10	1	2
Group A	10	10	1	2
Group A	10	10	1	2
Group A	10	10	1	2
Group A	10	10	1	2
Group A	10	10	1	2
Group A	10	10	1	2
Group A	10	10	1	2
Group A	10	10	2	1
Group A	10	10	2	1
Group A	10	10	2	1
Group A	10	10	2	1
Group A	10	10	2	1
Group A	10	10	2	1
Group A	10	10	2	1
Group A	10	10	2	1
Group A	10	10	2	1
Group A	10	10	2	1
Group A	10	10	2	2
Group A	10	10	2	2
Group A	10	10	2	2
Group A	10	10	2	2
Group A	10	10	2	2
Group A	10	10	2	2
Group A	10	10	2	2
Group A	10	10	2	2
Group A	10	10	2	2
Group A	10	10	2	2
Group A	10	10	3	1
Group A	10	10	3	1
Group A	10	10	3	1
Group A	10	10	3	1
Group A	10	10	3	1
Group A	10	10	3	1
Group A	10	10	3	1
Group A	10	10	3	1
Group A	10	10	3	1
Group A	10	10	3	1
Group A	10	10	3	2
Group A	10	10	3	2
Group A	10	10	3	2
Group A	10	10	3	2
Group A	10	10	3	2
Group A	10	10	3	2
Group A	10	10	3	2
Group A	10	10	3	2
Group A	10	10	3	2
Group A	10	10	3	2
Group A	10	10	4	1
Group A	10	10	4	1
Group A	10	10	4	1
Group A	10	10	4	1
Group A	10	10	4	1
Group A	10	10	4	1
Group A	10	10	4	1
Group A	10	10	4	1
Group A	10	10	4	1
Group A	10	10	4	1
Group A	10	10	4	2
Group A	10	10	4	2
Group A	10	10	4	2
Group A	10	10	4	2
Group A	10	10	4	2
Group A	10	10	4	2
Group A	10	10	4	2
Group A	10	10	4	2
Group A	10	10	4	2
Group A	10	10	4	2
Group A	10	10	5	1
Group A	10	10	5	1
Group A	10	10	5	1
Group A	10	10	5	1
Group A	10	10	5	1
Group A	10	10	5	1
Group A	10	10	5	1
Group A	10	10	5	1
Group A	10	10	5	1
Group A	10	10	5	1
Group A	10	10	5	2
Group A	10	10	5	2
Group A	10	10	5	2
Group A	10	10	5	2
Group A	10	10	5	2
Group A	10	10	5	2
Group A	10	10	5	2
Group A	10	10	5	2
Group A	10	10	5	2
Group A	10	10	5	2
Group A	10	10	6	1
Group A	10	10	6	1
Group A	10	10	6	1
Group A	10	10	6	1
Group A	10	10	6	1
Group A	10	10	6	1
Group A	10	10	6	1
Group A	10	10	6	1
Group A	10	10	6	1
Group A	10	10	6	1
Group A	10	10	6	2
Group A	10	10	6	2
Group A	10	10	6	2
Group A	10	10	6	2
Group A	10	10	6	2
Group A	10	10	6	2
Group A	10	10	6	2
Group A	10	10	6	2
Group A	10	10	6	2
Group A	10	10	6	2

B

PCR	Samples tested	samples correctly identified	Day	Technician
Group B	10	10	1	1
Group B	10	10	1	1
Group B	10	10	1	1
Group B	10	10	1	1
Group B	10	10	1	1
Group B	10	10	1	1
Group B	10	10	1	1
Group B	10	10	1	1
Group B	10	10	1	1
Group B	10	10	1	1
Group B	10	10	1	2
Group B	10	10	1	2
Group B	10	10	1	2
Group B	10	10	1	2
Group B	10	10	1	2
Group B	10	10	1	2
Group B	10	10	1	2
Group B	10	10	1	2
Group B	10	10	1	2
Group B	10	10	1	2
Group B	10	10	2	1
Group B	10	10	2	1
Group B	10	10	2	1
Group B	10	10	2	1
Group B	10	10	2	1
Group B	10	10	2	1
Group B	10	10	2	1
Group B	10	10	2	1
Group B	10	10	2	1
Group B	10	10	2	1
Group B	10	10	2	2
Group B	10	10	2	2
Group B	10	10	2	2
Group B	10	10	2	2
Group B	10	10	2	2
Group B	10	10	2	2
Group B	10	10	2	2
Group B	10	10	2	2
Group B	10	10	2	2
Group B	10	10	2	2
Group B	10	10	3	1
Group B	10	10	3	1
Group B	10	10	3	1
Group B	10	10	3	1
Group B	10	10	3	1
Group B	10	10	3	1
Group B	10	10	3	1
Group B	10	10	3	1
Group B	10	10	3	1
Group B	10	10	3	1
Group B	10	10	3	2
Group B	10	10	3	2
Group B	10	10	3	2
Group B	10	10	3	2
Group B	10	10	3	2
Group B	10	10	3	2
Group B	10	10	3	2
Group B	10	10	3	2
Group B	10	10	3	2
Group B	10	10	3	2
Group B	10	10	4	1
Group B	10	10	4	1
Group B	10	10	4	1
Group B	10	10	4	1
Group B	10	10	4	1
Group B	10	10	4	1
Group B	10	10	4	1
Group B	10	10	4	1
Group B	10	10	4	1
Group B	10	10	4	1
Group B	10	10	4	2
Group B	10	10	4	2
Group B	10	10	4	2
Group B	10	10	4	2
Group B	10	10	4	2
Group B	10	10	4	2
Group B	10	10	4	2
Group B	10	10	4	2
Group B	10	10	4	2
Group B	10	10	4	2
Group B	10	10	5	1
Group B	10	10	5	1
Group B	10	10	5	1
Group B	10	10	5	1
Group B	10	10	5	1
Group B	10	10	5	1
Group B	10	10	5	1
Group B	10	10	5	1
Group B	10	10	5	1
Group B	10	10	5	1
Group B	10	10	5	2
Group B	10	10	5	2
Group B	10	10	5	2
Group B	10	10	5	2
Group B	10	10	5	2
Group B	10	10	5	2
Group B	10	10	5	2
Group B	10	10	5	2
Group B	10	10	5	2
Group B	10	10	5	2
Group B	10	10	6	1
Group B	10	10	6	1
Group B	10	10	6	1
Group B	10	10	6	1
Group B	10	10	6	1
Group B	10	10	6	1
Group B	10	10	6	1
Group B	10	10	6	1
Group B	10	10	6	1
Group B	10	10	6	1
Group B	10	10	6	2
Group B	10	10	6	2
Group B	10	10	6	2
Group B	10	10	6	2
Group B	10	10	6	2
Group B	10	10	6	2
Group B	10	10	6	2
Group B	10	10	6	2
Group B	10	10	6	2
Group B	10	10	6	2

C

PCR	Samples tested	samples correctly identified	Day	Technician
Group C	10	10	1	1
Group C	10	10	1	1
Group C	10	10	1	1
Group C	10	10	1	1
Group C	10	10	1	1
Group C	10	10	1	1
Group C	10	10	1	1
Group C	10	10	1	1
Group C	10	10	1	1
Group C	10	10	1	1
Group C	10	10	1	2
Group C	10	10	1	2
Group C	10	10	1	2
Group C	10	10	1	2
Group C	10	10	1	2
Group C	10	10	1	2
Group C	10	10	1	2
Group C	10	10	1	2
Group C	10	10	1	2
Group C	10	10	1	2
Group C	10	10	2	1
Group C	10	10	2	1
Group C	10	10	2	1
Group C	10	10	2	1
Group C	10	10	2	1
Group C	10	10	2	1
Group C	10	10	2	1
Group C	10	10	2	1
Group C	10	10	2	1
Group C	10	10	2	1
Group C	10	10	2	2
Group C	10	10	2	2
Group C	10	10	2	2
Group C	10	10	2	2
Group C	10	10	2	2
Group C	10	10	2	2
Group C	10	10	2	2
Group C	10	10	2	2
Group C	10	10	2	2
Group C	10	10	2	2
Group C	10	10	3	1
Group C	10	10	3	1
Group C	10	10	3	1
Group C	10	10	3	1
Group C	10	10	3	1
Group C	10	10	3	1
Group C	10	10	3	1
Group C	10	10	3	1
Group C	10	10	3	1
Group C	10	10	3	1
Group C	10	10	3	2
Group C	10	10	3	2
Group C	10	10	3	2
Group C	10	10	3	2
Group C	10	10	3	2
Group C	10	10	3	2
Group C	10	10	3	2
Group C	10	10	3	2
Group C	10	10	3	2
Group C	10	10	3	2
Group C	10	10	4	1
Group C	10	10	4	1
Group C	10	10	4	1
Group C	10	10	4	1
Group C	10	10	4	1
Group C	10	10	4	1
Group C	10	10	4	1
Group C	10	10	4	1
Group C	10	10	4	1
Group C	10	10	4	1
Group C	10	10	4	2
Group C	10	10	4	2
Group C	10	10	4	2
Group C	10	10	4	2
Group C	10	10	4	2
Group C	10	10	4	2
Group C	10	10	4	2
Group C	10	10	4	2
Group C	10	10	4	2
Group C	10	10	4	2
Group C	10	10	5	1
Group C	10	10	5	1
Group C	10	10	5	1
Group C	10	10	5	1
Group C	10	10	5	1
Group C	10	10	5	1
Group C	10	10	5	1
Group C	10	10	5	1
Group C	10	10	5	1
Group C	10	10	5	1
Group C	10	10	5	2
Group C	10	10	5	2
Group C	10	10	5	2
Group C	10	10	5	2
Group C	10	10	5	2
Group C	10	10	5	2
Group C	10	10	5	2
Group C	10	10	5	2
Group C	10	10	5	2
Group C	10	10	5	2
Group C	10	10	6	1
Group C	10	10	6	1
Group C	10	10	6	1
Group C	10	10	6	1
Group C	10	10	6	1
Group C	10	10	6	1
Group C	10	10	6	1
Group C	10	10	6	1
Group C	10	10	6	1
Group C	10	10	6	1
Group C	10	10	6	2
Group C	10	10	6	2
Group C	10	10	6	2
Group C	10	10	6	2
Group C	10	10	6	2
Group C	10	10	6	2
Group C	10	10	6	2
Group C	10	10	6	2
Group C	10	10	6	2
Group C	10	10	6	2

3.4.2 Determination of the cut off value

The cut off value of the three *S. suis* assays was determined. Since the identity test was based on results of samples taken from several colonies (N=10) after culture on agar plates, the tolerance interval covering with a confidence of 99% a proportion of 99% of the Ct results was calculated.

The data used for the calculations are shown in addendum 1. Results of the distribution analysis are presented in addendum 2 and Figure 2 for *S. suis* group A, Figure 3 for *S. suis* group B, Figure 4 for *S. suis* group C.

The tolerance interval limits are summarized in Table 5 for *S. suis* group A, Table 6 for *S. suis* group B, Table 7 for *S. suis* group C. The upper limit of the tolerance interval was used as upper specification limit and the estimated and observed percentage of the Ct values above this upper specification level are provided in Addendum 2 and Table 5-7.

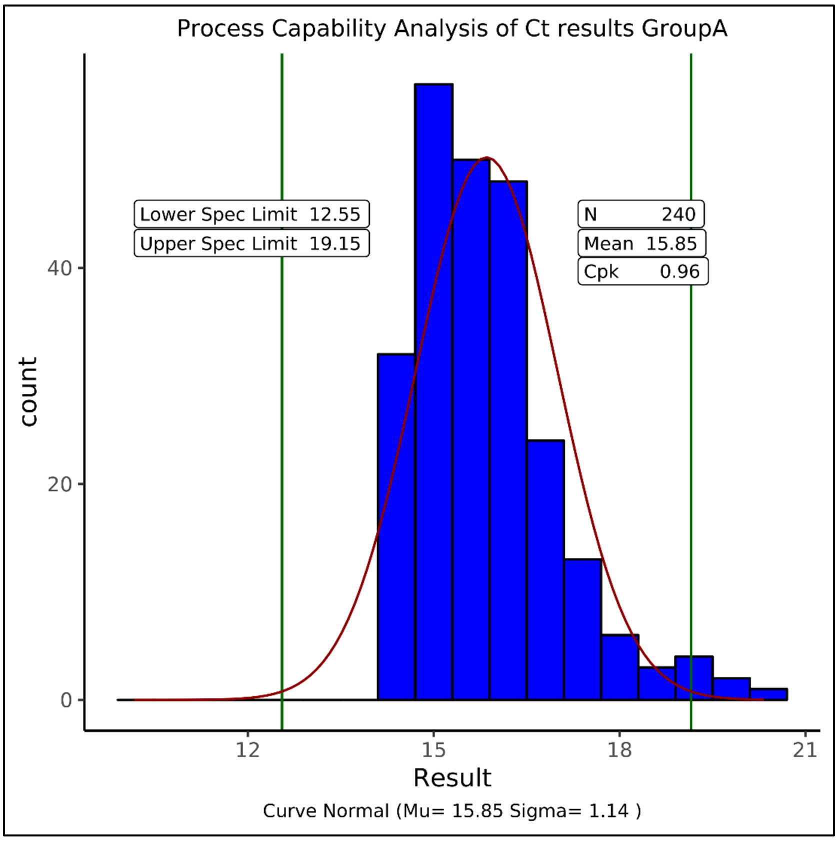

Figure 2. *S. suis* Group A.

Table 5. Estimated tolerance interval covering with a confidence of 99%, a proportion of 99% of the measurements (see addendum 2 Group A).

PCR	Mean Ct (sd)	Tolerance interval (confidence 99% proportion 99%		Percentage > upper limit tolerance interval (>usl)[a]		Cpk value for set usl as cut-off
		lower limit	upper limit	Observed (%)	Estimated (%)[b]	
S. suis group A	15.85 (1.14)	12.55	19.15	2.08	0.19	0.96

[a] upper specification, [b] based on normal distribution of Ct values.

The cut off for this test was set to Ct=20.0 (99% upper level was 19.15), as can be seen in Table 5. Using the upper limit of the tolerance interval covering with confidence of 99% a proportion of 99% of the Ct values, as cut-off has an acceptable risk of testing negative, providing that the Ct values of real negative samples are in the order of 3 standard deviation above this upper limit. The Cpk (assay performance index) value is close to a value 1 which is minimal desired.

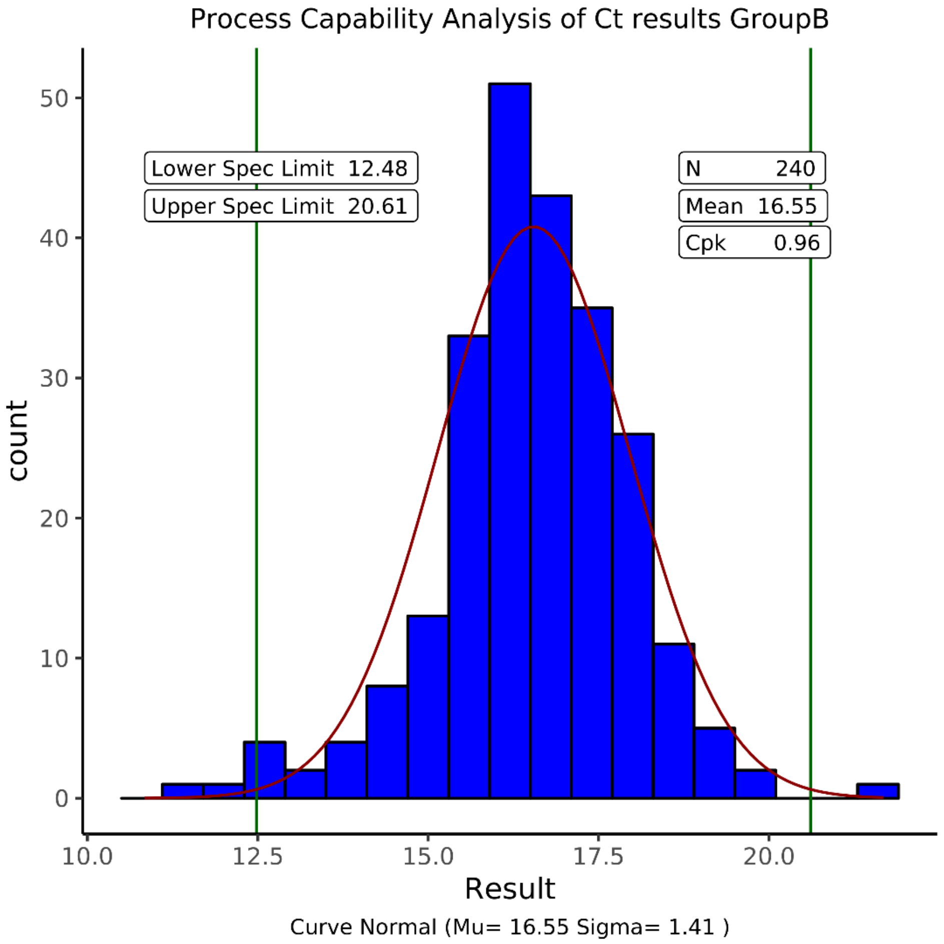

Figure 3. ***S. suis*** **Group B.**

Table 6. Estimated tolerance interval covering with a confidence of 99%, a proportion of 99% of the measurements (see addendum 2, Group B).

PCR	Mean Ct (sd)	Tolerance interval (confidence 99% proportion 99%		Percentage > upper limit tolerance interval (>usl)[a]		Cpk value for set usl as cut-off
		lower limit	upper limit	Observed (%)	Estimated (%)[b]	
S. suis group B	16.55 (1.41)	12.48	20.61	0.41	0.19	0.96

[a] upper specification, [b] based on normal distribution of Ct values.

The cut off for this test was set to Ct=21.0 (99% upper level was 20.61), as can be seen in Table 6. Using the upper limit of the tolerance interval covering with confidence of 99% a proportion of 99% of the Ct values, as cut-off has an acceptable risk of testing negative, providing that the Ct values of real negative samples are in the order of 3 standard deviation above this upper limit. The Cpk (assay performance index) value is close to a value 1 which is minimal desired.

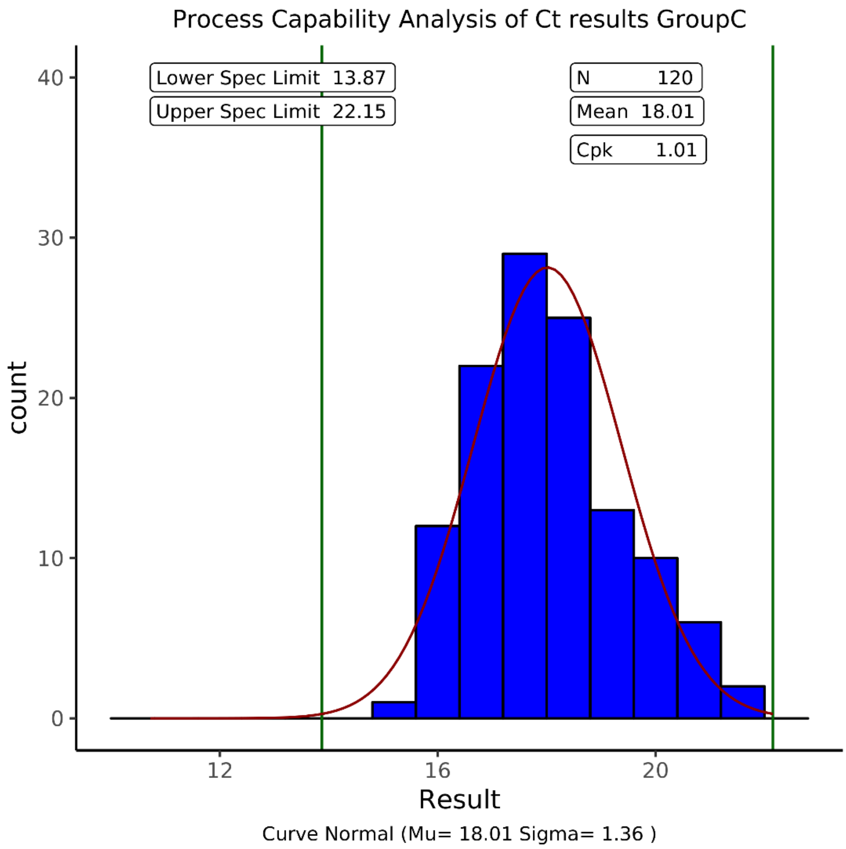

Figure 4. ***S. suis*** **group C.**

Table 7. Estimated tolerance interval covering with a confidence of 99%, a proportion of 99% of the measurements (see addendum 2 ***S. suis*** **group C).**

PCR	Mean Ct (sd)	Tolerance interval (confidence 99% proportion 99%		Percentage > upper limit tolerance interval (>usl)[a]		Cpk value for set usl as cut-off
		lower limit	upper limit	Observed (%)	Estimated (%)[b]	
S. suis group C	18.01 (1.36)	13.87	22.15	0	0.12	1.01

[a] upper specification, [b] based on normal distribution of Ct values

The cut off for this test was set to Ct=23.0 (99% upper level was 22.15), as can be seen in Table 7. Using the upper limit of the tolerance interval covering with confidence of 99% a proportion of 99% of the Ct values, as cut-off has an acceptable risk of testing negative, providing that the Ct values of real negative samples are in the order of 3 standard deviation above this upper limit. The Cpk (assay performance index) value is close to a value 1 which is minimal desired.

4. Conclusions

The qPCR assay showed no matrix effect and both repeatability and intermediate precision was determined for the method and samples were correctly identified in 6 different runs by 2 technicians on 10 different colonies. The cut off value of the three assays *S. suis* group A, *S. suis* group B and *S. suis* group C were calculated and set to Ct=20, Ct=21 and Ct=23.0, respectively, with the distribution analysis and estimated tolerance interval.

Addendum 1. Precision data

Precision data group A

PCR	Technician	Day	Colony	Sample	Result
GroupA	1	1	A1	1	16,32
GroupA	1	1	A1	2	18,86
GroupA	1	1	A1	3	16,31
GroupA	1	1	A1	4	16,49
GroupA	1	1	A1	5	16,31
GroupA	1	1	A1	6	18,63
GroupA	1	1	A1	7	17,03
GroupA	1	1	A1	8	17,50
GroupA	1	1	A1	9	16,77
GroupA	1	1	A1	10	16,17
GroupA	1	1	A2	1	18,95
GroupA	1	1	A2	2	16,28
GroupA	1	1	A2	3	16,50
GroupA	1	1	A2	4	15,29
GroupA	1	1	A2	5	14,83
GroupA	1	1	A2	6	16,07
GroupA	1	1	A2	7	14,96
GroupA	1	1	A2	8	16,02
GroupA	1	1	A2	9	15,59
GroupA	1	1	A2	10	14,76
GroupA	2	1	A1	1	18,63
GroupA	2	1	A1	2	16,96
GroupA	2	1	A1	3	18,99
GroupA	2	1	A1	4	19,42
GroupA	2	1	A1	5	19,65
GroupA	2	1	A1	6	16,17
GroupA	2	1	A1	7	16,64
GroupA	2	1	A1	8	16,63
GroupA	2	1	A1	9	17,33
GroupA	2	1	A1	10	16,60
GroupA	2	1	A2	1	16,04
GroupA	2	1	A2	2	15,85
GroupA	2	1	A2	3	14,29
GroupA	2	1	A2	4	14,72
GroupA	2	1	A2	5	14,93
GroupA	2	1	A2	6	14,67
GroupA	2	1	A2	7	15,22
GroupA	2	1	A2	8	15,13
GroupA	2	1	A2	9	15,23
GroupA	2	1	A2	10	14,73
GroupA	1	2	A1	1	17,64
GroupA	1	2	A1	2	15,74
GroupA	1	2	A1	3	15,93
GroupA	1	2	A1	4	16,65
GroupA	1	2	A1	5	15,78
GroupA	1	2	A1	6	15,81
GroupA	1	2	A1	7	15,94
GroupA	1	2	A1	8	16,50
GroupA	1	2	A1	9	16,10
GroupA	1	2	A1	10	16,52
GroupA	1	2	A2	1	17,96
GroupA	1	2	A2	2	15,74

GroupA	1	2	A2	3	16,05
GroupA	1	2	A2	4	16,07
GroupA	1	2	A2	5	15,56
GroupA	1	2	A2	6	14,85
GroupA	1	2	A2	7	15,33
GroupA	1	2	A2	8	16,45
GroupA	1	2	A2	9	14,89
GroupA	1	2	A2	10	15,85
GroupA	2	2	A1	1	16,25
GroupA	2	2	A1	2	16,17
GroupA	2	2	A1	3	17,18
GroupA	2	2	A1	4	16,42
GroupA	2	2	A1	5	17,49
GroupA	2	2	A1	6	15,55
GroupA	2	2	A1	7	18,23
GroupA	2	2	A1	8	16,37
GroupA	2	2	A1	9	17,78
GroupA	2	2	A1	10	15,81
GroupA	2	2	A2	1	14,35
GroupA	2	2	A2	2	14,26
GroupA	2	2	A2	3	14,50
GroupA	2	2	A2	4	14,43
GroupA	2	2	A2	5	14,15
GroupA	2	2	A2	6	16,48
GroupA	2	2	A2	7	14,43
GroupA	2	2	A2	8	14,67
GroupA	2	2	A2	9	16,60
GroupA	2	2	A2	10	14,41
GroupA	1	3	A1	1	15,14
GroupA	1	3	A1	2	14,72
GroupA	1	3	A1	3	14,44
GroupA	1	3	A1	4	15,59
GroupA	1	3	A1	5	15,51
GroupA	1	3	A1	6	15,89
GroupA	1	3	A1	7	15,51
GroupA	1	3	A1	8	15,57
GroupA	1	3	A1	9	14,98
GroupA	1	3	A1	10	15,11
GroupA	1	3	A2	1	16,45
GroupA	1	3	A2	2	16,61
GroupA	1	3	A2	3	15,98
GroupA	1	3	A2	4	16,07
GroupA	1	3	A2	5	17,25
GroupA	1	3	A2	6	15,69
GroupA	1	3	A2	7	16,51
GroupA	1	3	A2	8	16,23
GroupA	1	3	A2	9	16,15
GroupA	1	3	A2	10	17,12
GroupA	2	3	A1	1	15,92
GroupA	2	3	A1	2	16,81
GroupA	2	3	A1	3	15,57
GroupA	2	3	A1	4	17,30
GroupA	2	3	A1	5	17,84
GroupA	2	3	A1	6	16,97
GroupA	2	3	A1	7	14,91
GroupA	2	3	A1	8	17,89

GroupA	2	3	A1	9	16,47
GroupA	2	3	A1	10	15,33
GroupA	2	3	A2	1	16,31
GroupA	2	3	A2	2	16,62
GroupA	2	3	A2	3	16,80
GroupA	2	3	A2	4	19,51
GroupA	2	3	A2	5	18,27
GroupA	2	3	A2	6	16,52
GroupA	2	3	A2	7	17,53
GroupA	2	3	A2	8	20,33
GroupA	2	3	A2	9	17,65
GroupA	2	3	A2	10	19,22
GroupA	1	4	A1	1	14,76
GroupA	1	4	A1	2	15,53
GroupA	1	4	A1	3	14,75
GroupA	1	4	A1	4	14,69
GroupA	1	4	A1	5	15,66
GroupA	1	4	A1	6	14,75
GroupA	1	4	A1	7	14,69
GroupA	1	4	A1	8	15,40
GroupA	1	4	A1	9	15,54
GroupA	1	4	A1	10	15,30
GroupA	1	4	A2	1	15,36
GroupA	1	4	A2	2	14,97
GroupA	1	4	A2	3	15,69
GroupA	1	4	A2	4	14,67
GroupA	1	4	A2	5	15,89
GroupA	1	4	A2	6	15,26
GroupA	1	4	A2	7	15,73
GroupA	1	4	A2	8	16,53
GroupA	1	4	A2	9	16,95
GroupA	1	4	A2	10	15,01
GroupA	2	4	A1	1	14,80
GroupA	2	4	A1	2	15,32
GroupA	2	4	A1	3	15,47
GroupA	2	4	A1	4	14,98
GroupA	2	4	A1	5	15,16
GroupA	2	4	A1	6	14,97
GroupA	2	4	A1	7	14,62
GroupA	2	4	A1	8	16,91
GroupA	2	4	A1	9	14,80
GroupA	2	4	A1	10	15,15
GroupA	2	4	A2	1	15,61
GroupA	2	4	A2	2	15,05
GroupA	2	4	A2	3	15,25
GroupA	2	4	A2	4	16,58
GroupA	2	4	A2	5	14,96
GroupA	2	4	A2	6	15,77
GroupA	2	4	A2	7	16,05
GroupA	2	4	A2	8	17,56
GroupA	2	4	A2	9	14,94
GroupA	2	4	A2	10	15,91
GroupA	1	5	A1	1	14,66
GroupA	1	5	A1	2	14,22
GroupA	1	5	A1	3	14,21
GroupA	1	5	A1	4	14,79

Final /148500 / 1.0

GroupA	1	5	A1	5	14,43
GroupA	1	5	A1	6	14,39
GroupA	1	5	A1	7	14,63
GroupA	1	5	A1	8	15,39
GroupA	1	5	A1	9	15,09
GroupA	1	5	A1	10	15,17
GroupA	1	5	A2	1	14,54
GroupA	1	5	A2	2	14,70
GroupA	1	5	A2	3	15,64
GroupA	1	5	A2	4	15,01
GroupA	1	5	A2	5	16,71
GroupA	1	5	A2	6	16,45
GroupA	1	5	A2	7	15,86
GroupA	1	5	A2	8	15,63
GroupA	1	5	A2	9	15,99
GroupA	1	5	A2	10	15,19
GroupA	2	5	A1	1	14,68
GroupA	2	5	A1	2	14,55
GroupA	2	5	A1	3	14,76
GroupA	2	5	A1	4	14,91
GroupA	2	5	A1	5	14,76
GroupA	2	5	A1	6	14,72
GroupA	2	5	A1	7	14,67
GroupA	2	5	A1	8	14,48
GroupA	2	5	A1	9	14,73
GroupA	2	5	A1	10	14,94
GroupA	2	5	A2	1	15,63
GroupA	2	5	A2	2	15,74
GroupA	2	5	A2	3	15,78
GroupA	2	5	A2	4	15,48
GroupA	2	5	A2	5	15,22
GroupA	2	5	A2	6	15,00
GroupA	2	5	A2	7	15,90
GroupA	2	5	A2	8	16,24
GroupA	2	5	A2	9	15,25
GroupA	2	5	A2	10	15,56
GroupA	1	6	A1	1	15,88
GroupA	1	6	A1	2	16,37
GroupA	1	6	A1	3	16,00
GroupA	1	6	A1	4	16,34
GroupA	1	6	A1	5	16,91
GroupA	1	6	A1	6	16,41
GroupA	1	6	A1	7	15,96
GroupA	1	6	A1	8	15,27
GroupA	1	6	A1	9	15,56
GroupA	1	6	A1	10	16,00
GroupA	1	6	A2	1	15,44
GroupA	1	6	A2	2	16,29
GroupA	1	6	A2	3	14,97
GroupA	1	6	A2	4	15,36
GroupA	1	6	A2	5	16,89
GroupA	1	6	A2	6	15,80
GroupA	1	6	A2	7	16,38
GroupA	1	6	A2	8	15,08
GroupA	1	6	A2	9	17,41
GroupA	1	6	A2	10	15,14

Final /148500 / 1.0

GroupA	2	6	A1	1	14,28
GroupA	2	6	A1	2	14,44
GroupA	2	6	A1	3	15,08
GroupA	2	6	A1	4	17,41
GroupA	2	6	A1	5	15,05
GroupA	2	6	A1	6	16,01
GroupA	2	6	A1	7	16,15
GroupA	2	6	A1	8	15,22
GroupA	2	6	A1	9	15,75
GroupA	2	6	A1	10	15,51
GroupA	2	6	A2	1	14,27
GroupA	2	6	A2	2	14,31
GroupA	2	6	A2	3	15,41
GroupA	2	6	A2	4	15,32
GroupA	2	6	A2	5	14,72
GroupA	2	6	A2	6	15,23
GroupA	2	6	A2	7	15,99
GroupA	2	6	A2	8	16,15
GroupA	2	6	A2	9	16,91
GroupA	2	6	A2	10	14,42

Precision data group B

PCR	Technician	Day	Colony	Sample	Result
GroupB	1	1	B1	1	17,22
GroupB	1	1	B1	2	17,38
GroupB	1	1	B1	3	16,98
GroupB	1	1	B1	4	17,10
GroupB	1	1	B1	5	18,27
GroupB	1	1	B1	6	19,16
GroupB	1	1	B1	7	17,73
GroupB	1	1	B1	8	18,26
GroupB	1	1	B1	9	17,33
GroupB	1	1	B1	10	17,59
GroupB	1	1	B2	1	21,68
GroupB	1	1	B2	2	19,35
GroupB	1	1	B2	3	18,96
GroupB	1	1	B2	4	19,23
GroupB	1	1	B2	5	18,88
GroupB	1	1	B2	6	18,52
GroupB	1	1	B2	7	17,47
GroupB	1	1	B2	8	17,73
GroupB	1	1	B2	9	16,72
GroupB	1	1	B2	10	18,24
GroupB	2	1	B1	1	13,70
GroupB	2	1	B1	2	13,69
GroupB	2	1	B1	3	13,49
GroupB	2	1	B1	4	14,51
GroupB	2	1	B1	5	15,52
GroupB	2	1	B1	6	16,58
GroupB	2	1	B1	7	15,18
GroupB	2	1	B1	8	12,60
GroupB	2	1	B1	9	15,89
GroupB	2	1	B1	10	16,72
GroupB	2	1	B2	1	14,96
GroupB	2	1	B2	2	11,42

GroupB	2	1	B2	3	12,44
GroupB	2	1	B2	4	14,03
GroupB	2	1	B2	5	12,20
GroupB	2	1	B2	6	12,55
GroupB	2	1	B2	7	12,54
GroupB	2	1	B2	8	13,06
GroupB	2	1	B2	9	15,75
GroupB	2	1	B2	10	15,02
GroupB	1	1	B1	1	18,72
GroupB	1	2	B1	2	16,79
GroupB	1	2	B1	3	16,84
GroupB	1	2	B1	4	16,34
GroupB	1	2	B1	5	16,78
GroupB	1	2	B1	6	15,76
GroupB	1	2	B1	7	17,48
GroupB	1	2	B1	8	17,71
GroupB	1	2	B1	9	17,20
GroupB	1	2	B1	10	16,63
GroupB	1	2	B2	1	16,51
GroupB	1	2	B2	2	15,91
GroupB	1	2	B2	3	16,22
GroupB	1	2	B2	4	16,81
GroupB	1	2	B2	5	16,40
GroupB	1	2	B2	6	16,23
GroupB	1	2	B2	7	16,79
GroupB	1	2	B2	8	16,48
GroupB	1	2	B2	9	16,50
GroupB	1	2	B2	10	15,97
GroupB	2	2	B1	1	16,40
GroupB	2	2	B1	2	15,68
GroupB	2	2	B1	3	18,07
GroupB	2	2	B1	4	17,13
GroupB	2	2	B1	5	18,30
GroupB	2	2	B1	6	16,99
GroupB	2	2	B1	7	17,84
GroupB	2	2	B1	8	17,94
GroupB	2	2	B1	9	18,22
GroupB	2	2	B1	10	16,86
GroupB	2	2	B2	1	17,35
GroupB	2	2	B2	2	16,00
GroupB	2	2	B2	3	18,51
GroupB	2	2	B2	4	17,66
GroupB	2	2	B2	5	15,77
GroupB	2	2	B2	6	17,22
GroupB	2	2	B2	7	18,08
GroupB	2	2	B2	8	16,56
GroupB	2	2	B2	9	18,43
GroupB	2	2	B2	10	17,19
GroupB	1	3	B1	1	16,31
GroupB	1	3	B1	2	16,59
GroupB	1	3	B1	3	17,38
GroupB	1	3	B1	4	16,74
GroupB	1	3	B1	5	17,51
GroupB	1	3	B1	6	15,87
GroupB	1	3	B1	7	17,54
GroupB	1	3	B1	8	16,81

GroupB	1	3	B1	9	16,75
GroupB	1	3	B1	10	16,33
GroupB	1	3	B2	1	16,22
GroupB	1	3	B2	2	15,94
GroupB	1	3	B2	3	15,31
GroupB	1	3	B2	4	14,32
GroupB	1	3	B2	5	15,78
GroupB	1	3	B2	6	15,00
GroupB	1	3	B2	7	14,99
GroupB	1	3	B2	8	15,78
GroupB	1	3	B2	9	16,42
GroupB	1	3	B2	10	15,35
GroupB	2	3	B1	1	16,42
GroupB	2	3	B1	2	15,60
GroupB	2	3	B1	3	17,62
GroupB	2	3	B1	4	16,52
GroupB	2	3	B1	5	15,83
GroupB	2	3	B1	6	15,71
GroupB	2	3	B1	7	17,59
GroupB	2	3	B1	8	15,93
GroupB	2	3	B1	9	17,75
GroupB	2	3	B1	10	15,52
GroupB	2	3	B2	1	16,95
GroupB	2	3	B2	2	17,25
GroupB	2	3	B2	3	14,70
GroupB	2	3	B2	4	17,07
GroupB	2	3	B2	5	17,04
GroupB	2	3	B2	6	18,82
GroupB	2	3	B2	7	15,30
GroupB	2	3	B2	8	16,16
GroupB	2	3	B2	9	15,84
GroupB	2	3	B2	10	18,76
GroupB	1	4	B1	1	15,95
GroupB	1	4	B1	2	16,93
GroupB	1	4	B1	3	16,12
GroupB	1	4	B1	4	16,82
GroupB	1	4	B1	5	15,99
GroupB	1	4	B1	6	17,44
GroupB	1	4	B1	7	16,11
GroupB	1	4	B1	8	16,12
GroupB	1	4	B1	9	16,98
GroupB	1	4	B1	10	16,58
GroupB	1	4	B2	1	15,07
GroupB	1	4	B2	2	15,93
GroupB	1	4	B2	3	16,71
GroupB	1	4	B2	4	15,99
GroupB	1	4	B2	5	15,43
GroupB	1	4	B2	6	15,83
GroupB	1	4	B2	7	15,56
GroupB	1	4	B2	8	16,19
GroupB	1	4	B2	9	16,10
GroupB	1	4	B2	10	15,79
GroupB	2	4	B1	1	15,96
GroupB	2	4	B1	2	16,94
GroupB	2	4	B1	3	17,64
GroupB	2	4	B1	4	17,90

Final /148500 / 1.0

GroupB	2	4	B1	5	17,12
GroupB	2	4	B1	6	16,56
GroupB	2	4	B1	7	16,90
GroupB	2	4	B1	8	17,09
GroupB	2	4	B1	9	17,19
GroupB	2	4	B1	10	18,25
GroupB	2	4	B2	1	17,35
GroupB	2	4	B2	2	17,40
GroupB	2	4	B2	3	17,21
GroupB	2	4	B2	4	17,04
GroupB	2	4	B2	5	18,07
GroupB	2	4	B2	6	17,35
GroupB	2	4	B2	7	16,65
GroupB	2	4	B2	8	17,71
GroupB	2	4	B2	9	16,46
GroupB	2	4	B2	10	18,54
GroupB	1	5	B1	1	18,55
GroupB	1	5	B1	2	18,05
GroupB	1	5	B1	3	18,57
GroupB	1	5	B1	4	17,31
GroupB	1	5	B1	5	17,57
GroupB	1	5	B1	6	18,25
GroupB	1	5	B1	7	19,74
GroupB	1	5	B1	8	14,96
GroupB	1	5	B1	9	15,41
GroupB	1	5	B1	10	15,88
GroupB	1	5	B2	1	16,38
GroupB	1	5	B2	2	16,64
GroupB	1	5	B2	3	15,94
GroupB	1	5	B2	4	16,25
GroupB	1	5	B2	5	16,46
GroupB	1	5	B2	6	18,11
GroupB	1	5	B2	7	16,47
GroupB	1	5	B2	8	16,57
GroupB	1	5	B2	9	16,92
GroupB	1	5	B2	10	16,94
GroupB	2	5	B1	1	14,42
GroupB	2	5	B1	2	14,61
GroupB	2	5	B1	3	15,56
GroupB	2	5	B1	4	14,07
GroupB	2	5	B1	5	14,98
GroupB	2	5	B1	6	14,61
GroupB	2	5	B1	7	15,39
GroupB	2	5	B1	8	14,45
GroupB	2	5	B1	9	15,18
GroupB	2	5	B1	10	16,25
GroupB	2	5	B2	1	15,05
GroupB	2	5	B2	2	15,44
GroupB	2	5	B2	3	15,90
GroupB	2	5	B2	4	15,63
GroupB	2	5	B2	5	14,43
GroupB	2	5	B2	6	15,27
GroupB	2	5	B2	7	16,56
GroupB	2	5	B2	8	16,08
GroupB	2	5	B2	9	17,21
GroupB	2	5	B2	10	18,25

GroupB	1	6	B1	1	16,22
GroupB	1	6	B1	2	18,21
GroupB	1	6	B1	3	18,47
GroupB	1	6	B1	4	17,65
GroupB	1	6	B1	5	17,84
GroupB	1	6	B1	6	17,79
GroupB	1	6	B1	7	16,37
GroupB	1	6	B1	8	19,53
GroupB	1	6	B1	9	18,98
GroupB	1	6	B1	10	17,08
GroupB	1	6	B2	1	17,56
GroupB	1	6	B2	2	18,27
GroupB	1	6	B2	3	16,06
GroupB	1	6	B2	4	16,03
GroupB	1	6	B2	5	17,35
GroupB	1	6	B2	6	15,95
GroupB	1	6	B2	7	16,76
GroupB	1	6	B2	8	16,90
GroupB	1	6	B2	9	17,31
GroupB	1	6	B2	10	16,13
GroupB	2	6	B1	1	15,42
GroupB	2	6	B1	2	16,05
GroupB	2	6	B1	3	15,65
GroupB	2	6	B1	4	14,78
GroupB	2	6	B1	5	15,98
GroupB	2	6	B1	6	16,09
GroupB	2	6	B1	7	15,61
GroupB	2	6	B1	8	15,89
GroupB	2	6	B1	9	16,41
GroupB	2	6	B1	10	16,35
GroupB	2	6	B2	1	15,35
GroupB	2	6	B2	2	16,48
GroupB	2	6	B2	3	17,82
GroupB	2	6	B2	4	17,69
GroupB	2	6	B2	5	17,40
GroupB	2	6	B2	6	16,50
GroupB	2	6	B2	7	15,38
GroupB	2	6	B2	8	15,93
GroupB	2	6	B2	9	16,65
GroupB	2	6	B2	10	16,45

Precision data group C

PCR	Technician	Day	Colony	Sample	Result
GroupC	1	1	C1	1	20,90
GroupC	1	1	C1	2	20,29
GroupC	1	1	C1	3	18,95
GroupC	1	1	C1	4	19,05
GroupC	1	1	C1	5	21,44
GroupC	1	1	C1	6	18,35
GroupC	1	1	C1	7	20,05
GroupC	1	1	C1	8	18,85
GroupC	1	1	C1	9	18,92
GroupC	1	1	C1	10	19,35
GroupC	2	1	C1	1	18,12
GroupC	2	1	C1	2	17,41
GroupC	2	1	C1	3	18,05

GroupC	2	1	C1	4	17,73
GroupC	2	1	C1	5	18,39
GroupC	2	1	C1	6	18,59
GroupC	2	1	C1	7	17,56
GroupC	2	1	C1	8	16,32
GroupC	2	1	C1	9	16,47
GroupC	2	1	C1	10	18,67
GroupC	1	2	C1	1	20,83
GroupC	1	2	C1	2	21,41
GroupC	1	2	C1	3	19,27
GroupC	1	2	C1	4	17,95
GroupC	1	2	C1	5	19,82
GroupC	1	2	C1	6	19,06
GroupC	1	2	C1	7	20,46
GroupC	1	2	C1	8	20,28
GroupC	1	2	C1	9	20,68
GroupC	1	2	C1	10	18,59
GroupC	2	2	C1	1	17,58
GroupC	2	2	C1	2	17,74
GroupC	2	2	C1	3	18,06
GroupC	2	2	C1	4	18,50
GroupC	2	2	C1	5	20,81
GroupC	2	2	C1	6	17,99
GroupC	2	2	C1	7	17,34
GroupC	2	2	C1	8	18,10
GroupC	2	2	C1	9	16,80
GroupC	2	2	C1	10	18,04
GroupC	1	3	C1	1	15,63
GroupC	1	3	C1	2	16,39
GroupC	1	3	C1	3	16,32
GroupC	1	3	C1	4	16,48
GroupC	1	3	C1	5	16,66
GroupC	1	3	C1	6	17,26
GroupC	1	3	C1	7	16,16
GroupC	1	3	C1	8	16,10
GroupC	1	3	C1	9	16,88
GroupC	1	3	C1	10	16,88
GroupC	2	3	C1	1	18,42
GroupC	2	3	C1	2	16,08
GroupC	2	3	C1	3	18,42
GroupC	2	3	C1	4	18,04
GroupC	2	3	C1	5	18,24
GroupC	2	3	C1	6	17,91
GroupC	2	3	C1	7	18,57
GroupC	2	3	C1	8	17,54
GroupC	2	3	C1	9	19,51
GroupC	2	3	C1	10	18,14
GroupC	1	4	C1	1	17,76
GroupC	1	4	C1	2	17,35
GroupC	1	4	C1	3	17,68
GroupC	1	4	C1	4	17,70
GroupC	1	4	C1	5	18,58
GroupC	1	4	C1	6	17,21
GroupC	1	4	C1	7	17,09
GroupC	1	4	C1	8	16,92
GroupC	1	4	C1	9	17,44

GroupC	1	4	C1	10	19,10
GroupC	2	4	C1	1	16,70
GroupC	2	4	C1	2	16,61
GroupC	2	4	C1	3	17,41
GroupC	2	4	C1	4	16,39
GroupC	2	4	C1	5	16,89
GroupC	2	4	C1	6	16,85
GroupC	2	4	C1	7	15,28
GroupC	2	4	C1	8	16,43
GroupC	2	4	C1	9	16,93
GroupC	2	4	C1	10	16,62
GroupC	1	5	C1	1	18,45
GroupC	1	5	C1	2	19,87
GroupC	1	5	C1	3	18,58
GroupC	1	5	C1	4	17,47
GroupC	1	5	C1	5	17,49
GroupC	1	5	C1	6	17,17
GroupC	1	5	C1	7	21,02
GroupC	1	5	C1	8	17,82
GroupC	1	5	C1	9	19,98
GroupC	1	5	C1	10	19,69
GroupC	2	5	C1	1	17,32
GroupC	2	5	C1	2	17,11
GroupC	2	5	C1	3	16,89
GroupC	2	5	C1	4	17,04
GroupC	2	5	C1	5	17,61
GroupC	2	5	C1	6	16,83
GroupC	2	5	C1	7	17,57
GroupC	2	5	C1	8	17,42
GroupC	2	5	C1	9	18,60
GroupC	2	5	C1	10	18,06
GroupC	1	6	C1	1	17,51
GroupC	1	6	C1	2	18,78
GroupC	1	6	C1	3	20,16
GroupC	1	6	C1	4	19,68
GroupC	1	6	C1	5	18,97
GroupC	1	6	C1	6	19,14
GroupC	1	6	C1	7	19,44
GroupC	1	6	C1	8	18,87
GroupC	1	6	C1	9	19,77
GroupC	1	6	C1	10	17,23
GroupC	2	6	C1	1	15,84
GroupC	2	6	C1	2	16,18
GroupC	2	6	C1	3	15,70
GroupC	2	6	C1	4	16,87
GroupC	2	6	C1	5	17,29
GroupC	2	6	C1	6	16,18
GroupC	2	6	C1	7	18,30
GroupC	2	6	C1	8	17,36
GroupC	2	6	C1	9	16,85
GroupC	2	6	C1	10	18,22

Addendum 2. Distribution analysis of the qPCR data

Addendum Group A

```
[1] "sigma/sd (table)"
[1] 1.143577
[1] "mu/mean (table)"
[1] 15.85179
[1] "interval"
  alpha    P    x.bar 2-sided.lower 2-sided.upper
1  0.01 0.99 15.85179      12.55004      19.15354
[1] "estimated %upper limit (table)"
[1] 0.1942167
[1] "observed %upper limit (table)"
[1] 2.083333
[1] "cpk (table)"
[1] 0.962404

[1] "Quantiles Estimated"
     1%      5%     10%     25%     50%     75%     90%     95%     99%
13.1914 13.9707 14.3862 15.0804 15.8517 16.6231 17.3173 17.7328 18.5121
[1] " Quantiles Observed"
     1%      5%     10%     25%     50%     75%     90%     95%     99%
14.2356 14.4295 14.6690 14.9775 15.6900 16.4500 17.3380 17.9735 19.4749
[1] "two sided statistical intervals tolerance interval"
[1] " Confidence 99% "
  alpha    P    x.bar 2-sided.lower 2-sided.upper
1  0.01 0.90 15.85179      13.74339      17.96020
2  0.01 0.95 15.85179      13.33947      18.36411
3  0.01 0.99 15.85179      12.55004      19.15354
[1] " Confidence 95% "
  alpha    P    x.bar 2-sided.lower 2-sided.upper
1  0.05 0.90 15.85179      13.81233      17.89126
2  0.05 0.95 15.85179      13.42162      18.28197
3  0.05 0.99 15.85179      12.65800      19.04558
[1] " Confidence 90% "
  alpha    P    x.bar 2-sided.lower 2-sided.upper
1   0.1 0.90 15.85179      13.84758      17.85601
2   0.1 0.95 15.85179      13.46362      18.23996
3   0.1 0.99 15.85179      12.71321      18.99038

[1] "anova"
Call:
   aov(formula = Result ~ as.factor(Technician) + as.factor(Day_tech),
    data = data)
```

```
Terms:
                as.factor(Technician) as.factor(Day_tech) Residuals
Sum of Squares                0.48690            82.40369 229.66593
Deg. of Freedom                     1                  10       228

Residual standard error: 1.003647
1 out of 13 effects not estimable
Estimated effects may be unbalanced
                       Df Sum Sq Mean Sq F value   Pr(>F)
as.factor(Technician)   1   0.49   0.487   0.483    0.488
as.factor(Day_tech)    10  82.40   8.240   8.181 2.72e-11 ***
Residuals             228 229.67   1.007
---
Signif. codes:  0 '***' 0.001 '**' 0.01 '*' 0.05 '.' 0.1 ' ' 1
```

```
[1] "model"
Linear mixed model fit by REML ['lmerModLmerTest']
Formula: Result ~ Technician + (1 | Day)
   Data: data
REML criterion at convergence: 715.9893
Random effects:
 Groups   Name        Std.Dev.
 Day      (Intercept) 0.521
 Residual             1.041
Number of obs: 240, groups:  Day, 6
Fixed Effects:
(Intercept)  Technician2
   15.80675      0.09008
[1] "anova"
Type III Analysis of Variance Table with Satterthwaite's method
           Sum Sq Mean Sq NumDF DenDF F value Pr(>F)
Technician 0.4869  0.4869     1   233  0.4495 0.5032
[1] "rand"
ANOVA-like table for random-effects: Single term deletions

Model:
Result ~ Technician + (1 | Day)
          npar  logLik    AIC    LRT Df Pr(>Chisq)
<none>       4 -357.99 723.99
(1 | Day)    3 -374.74 755.48 33.488  1  7.171e-09 ***
---
Signif. codes:  0 '***' 0.001 '**' 0.01 '*' 0.05 '.' 0.1 ' ' 1
[1] "difflsmeans"
Least Squares Means table:

                           Estimate Std. Error  df t value     lower     upper
Technician1 - Technician2 -0.090083   0.134356 233 -0.6705 -0.354792  0.174625
                          Pr(>|t|)
Technician1 - Technician2   0.5032

  Confidence level: 95%
  Degrees of freedom method: Satterthwaite
```

Addendum Group B

```
[1] "sigma/sd (table)"
[1] 1.407839
[1] "mu/mean (table)"
[1] 16.54525
[1] "interval"
  alpha    P    x.bar 2-sided.lower 2-sided.upper
1  0.01 0.99 16.54525      12.48052      20.60998
[1] "estimated %upper limit (table)"
[1] 0.1966833
[1] "observed %upper limit (table)"
[1] 0.4166667
[1] "cpk (table)"
[1] 0.962404

[1] "Quantiles Estimated"
     1%      5%     10%     25%     50%     75%     90%     95%     99%
13.2701 14.2296 14.7410 15.5957 16.5453 17.4948 18.3495 18.8609 19.8204
[1] "Quantiles Observed"
     1%      5%     10%     25%     50%     75%     90%     95%     99%
12.4790 14.3075 14.9890 15.8375 16.5600 17.4000 18.2500 18.5775 19.4598
[1] "two sided statistical intervals tolerance interval"
[1] " Confidence 99% "
  alpha    P    x.bar 2-sided.lower 2-sided.upper
1  0.01 0.90 16.54525      13.94962      19.14088
2  0.01 0.95 16.54525      13.45237      19.63813
3  0.01 0.99 16.54525      12.48052      20.60998
[1] " Confidence 95% "
  alpha    P    x.bar 2-sided.lower 2-sided.upper
1  0.05 0.90 16.54525      14.03449      19.05601
2  0.05 0.95 16.54525      13.55350      19.53700
3  0.05 0.99 16.54525      12.61343      20.47707
[1] " Confidence 90% "
  alpha    P    x.bar 2-sided.lower 2-sided.upper
1   0.1 0.90 16.54525      14.07789      19.01261
2   0.1 0.95 16.54525      13.60521      19.48529
3   0.1 0.99 16.54525      12.68139      20.40911
[1] "anova"

Call:
   aov(formula = Result ~ as.factor(Technician) + as.factor(Day_tech),
    data = data)

Terms:
```

```
                as.factor(Technician) as.factor(Day_tech) Residuals
Sum of Squares               35.96004          213.88113 223.85962
Deg. of Freedom                     1                 10       228

Residual standard error: 0.9908786
1 out of 13 effects not estimable
Estimated effects may be unbalanced
                       Df Sum Sq Mean Sq F value   Pr(>F)    
as.factor(Technician)   1  35.96   35.96   36.62 5.83e-09 ***
as.factor(Day_tech)    10 213.88   21.39   21.78  < 2e-16 ***
Residuals             228 223.86    0.98                     
---
Signif. codes:  0 '***' 0.001 '**' 0.01 '*' 0.05 '.' 0.1 ' ' 1
```

Final /148500 / 1.0

```
[1] "model"
Linear mixed model fit by REML ['lmerModLmerTest']
Formula: Result ~ Technician + (1 | Day)
   Data: data
REML criterion at convergence: 828.073
Random effects:
 Groups   Name        Std.Dev.
 Day      (Intercept) 0.2309
 Residual             1.3396
Number of obs: 240, groups:  Day, 6
Fixed Effects:
(Intercept)  Technician2
    16.9358      -0.7777
[1] "anova"
Type III Analysis of Variance Table with Satterthwaite's method
           Sum Sq Mean Sq NumDF  DenDF F value    Pr(>F)
Technician 36.283  36.283     1 233.03  20.219 1.089e-05 ***
---
Signif. codes:  0 '***' 0.001 '**' 0.01 '*' 0.05 '.' 0.1 ' ' 1
[1] "rand"
ANOVA-like table for random-effects: Single term deletions

Model:
Result ~ Technician + (1 | Day)
          npar  logLik    AIC    LRT Df Pr(>Chisq)
<none>       4 -414.04 836.07
(1 | Day)    3 -415.01 836.02 1.9435  1     0.1633
[1] "difflsmeans"
Least Squares Means table:

                           Estimate Std. Error  df t value   lower   upper
Technician1 - Technician2   0.77768    0.17295 233  4.4965 0.43694 1.11843
                            Pr(>|t|)
Technician1 - Technician2 1.089e-05 ***
---
Signif. codes:  0 '***' 0.001 '**' 0.01 '*' 0.05 '.' 0.1 ' ' 1

  Confidence level: 95%
  Degrees of freedom method: Satterthwaite
```

Addendum Group C

```
[1] "sigma/sd (table)"
[1] 1.360816
[1] "mu/mean (table)"
[1] 18.01392
[1] "interval"
  alpha    P    x.bar 2-sided.lower 2-sided.upper
1  0.01 0.99 18.01392       13.8731      22.15473
[1] "estimated %upper limit (table)"
[1] 0.1166083
[1] "observed %upper limit (table)"
[1] 0
[1] "cpk (table)"
[1] 1.014297

[1] "Quantiles Estimated"
     1%      5%     10%     25%     50%     75%     90%     95%     99%
14.8482 15.7756 16.2699 17.0961 18.0139 18.9318 19.7579 20.2523 21.1797
[1] "Quantiles Observed"
     1%      5%     10%     25%     50%     75%     90%     95%     99%
15.6433 16.1570 16.3900 16.9275 17.7900 18.8550 19.9870 20.6865 21.3359
[1] "two sided statistical intervals tolerance interval"
[1] " Confidence 99% "
  alpha    P    x.bar 2-sided.lower 2-sided.upper
1  0.01 0.90 18.01392      15.36971      20.65813
2  0.01 0.95 18.01392      14.86315      21.16469
3  0.01 0.99 18.01392      13.87310      22.15473
[1] " Confidence 95% "
  alpha    P    x.bar 2-sided.lower 2-sided.upper
1  0.05 0.90 18.01392      15.49487      20.53297
2  0.05 0.95 18.01392      15.01228      21.01555
3  0.05 0.99 18.01392      14.06910      21.95873
[1] " Confidence 90% "
  alpha    P    x.bar 2-sided.lower 2-sided.upper
1   0.1 0.90 18.01392      15.55769      20.47014
2   0.1 0.95 18.01392      15.08714      20.94069
3   0.1 0.99 18.01392      14.16748      21.86035
[1] "anova"
Call:
   aov(formula = Result ~ as.factor(Technician) + as.factor(Day_tech),
    data = data)

Terms:
                as.factor(Technician) as.factor(Day_tech) Residuals
```

```
Sum of Squares                 34.87330            99.72535  85.76801
Deg. of Freedom                       1                  10       108

Residual standard error: 0.89115
1 out of 13 effects not estimable
Estimated effects may be unbalanced
                       Df Sum Sq Mean Sq F value   Pr(>F)
as.factor(Technician)   1  34.87   34.87   43.91 1.39e-09 ***
as.factor(Day_tech)    10  99.73    9.97   12.56 3.07e-14 ***
Residuals             108  85.77    0.79
---
Signif. codes:  0 '***' 0.001 '**' 0.01 '*' 0.05 '.' 0.1 ' ' 1
```

Final /148500 / 1.0

```
[1] "model"
Linear mixed model fit by REML ['lmerModLmerTest']
Formula: Result ~ Technician + (1 | Day)
   Data: data
REML criterion at convergence: 372.9277
Random effects:
 Groups   Name        Std.Dev.
 Day      (Intercept) 0.6857
 Residual             1.0833
Number of obs: 120, groups:  Day, 6
Fixed Effects:
(Intercept)  Technician2
     18.553       -1.078
[1] "anova"
Type III Analysis of Variance Table with Satterthwaite's method
           Sum Sq Mean Sq NumDF DenDF F value    Pr(>F)
Technician 34.873  34.873     1   113  29.718 2.969e-07 ***
---
Signif. codes:  0 '***' 0.001 '**' 0.01 '*' 0.05 '.' 0.1 ' ' 1
[1] "rand"
ANOVA-like table for random-effects: Single term deletions

Model:
Result ~ Technician + (1 | Day)
          npar  logLik    AIC    LRT Df Pr(>Chisq)
<none>       4 -186.46 380.93
(1 | Day)    3 -198.22 402.43 23.506  1  1.245e-06 ***
---
Signif. codes:  0 '***' 0.001 '**' 0.01 '*' 0.05 '.' 0.1 ' ' 1
[1] "difflsmeans"
Least Squares Means table:

                          Estimate Std. Error  df t value   lower   upper
Technician1 - Technician2  1.07817    0.19778 113  5.4514 0.68634 1.47000
                           Pr(>|t|)
Technician1 - Technician2 2.969e-07 ***
---
Signif. codes:  0 '***' 0.001 '**' 0.01 '*' 0.05 '.' 0.1 ' ' 1

  Confidence level: 95%
  Degrees of freedom method: Satterthwaite
```

Final /148500 / 1.0
